# Excessive rainfall leads to maize yield loss of a comparable magnitude to extreme drought in the United States

**DOI:** 10.1111/gcb.14628

**Published:** 2019-04-29

**Authors:** Yan Li, Kaiyu Guan, Gary D. Schnitkey, Evan DeLucia, Bin Peng

**Affiliations:** ^1^ State Key Laboratory of Earth Surface Processes and Resources Ecology, Faculty of Geographical Science Beijing Normal University Beijing China; ^2^ Department of Natural Resources and Environmental Sciences University of Illinois at Urbana‐Champaign Urbana Illinois; ^3^ National Center for Supercomputing Applications University of Illinois at Urbana‐Champaign Urbana Illinois; ^4^ Department of Agricultural and Consumer Economics University of Illinois at Urbana‐Champaign Urbana Illinois; ^5^ Institute for Sustainability, Energy, and Environment University of Illinois at Urbana‐Champaign Urbana Illinois; ^6^ Department of Plant Biology University of Illinois at Urbana‐Champaign Urbana Illinois

**Keywords:** crop model, drought, extreme climate, extreme rainfall, maize production

## Abstract

Increasing drought and extreme rainfall are major threats to maize production in the United States. However, compared to drought impact, the impact of excessive rainfall on crop yield remains unresolved. Here, we present observational evidence from crop yield and insurance data that excessive rainfall can reduce maize yield up to −34% (−17 ± 3% on average) in the United States relative to the expected yield from the long‐term trend, comparable to the up to −37% loss by extreme drought (−32 ± 2% on average) from 1981 to 2016. Drought consistently decreases maize yield due to water deficiency and concurrent heat, with greater yield loss for rainfed maize in wetter areas. Excessive rainfall can have either negative or positive impact on crop yield, and its sign varies regionally. Excessive rainfall decreases maize yield significantly in cooler areas in conjunction with poorly drained soils, and such yield loss gets exacerbated under the condition of high preseason soil water storage. Current process‐based crop models cannot capture the yield loss from excessive rainfall and overestimate yield under wet conditions. Our results highlight the need for improved understanding and modeling of the excessive rainfall impact on crop yield.

## Graphical abstract

This study reveals that excessive rainfall can adversely affect maize yield as much as extreme drought in the US, especially at regional scale. However, current process‐based crop models cannot capture the yield loss from excessive rainfall and overestimate crop yield under wet conditions. The results highlight the need for improved understanding and modeling of the excessive rainfall impact on crop yield.

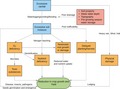

## 1 INTRODUCTION

Agricultural production and food security are threatened by the increases in temperature and precipitation extremes under a warming climate with intensified water cycle (Bengtsson, 2010; Chou et al., 2013; Hegerl et al., 2015; Lesk, Rowhani, & Ramankutty, 2016). The United States, the world's largest maize producer that supplies >30% of global maize production, has experienced significant increases of heat, drought, and extreme rainfall since 1980 (Groisman et al., 2005; Kunkel & Easterling, 1999; Mazdiyasni & AghaKouchak, 2015; Wuebbles et al., 2017). These climate extremes have already caused substantial damage to maize production in the United States (Lobell et al., 2013,2014; Pielke & Downton, 2000; Schlenker & Roberts, 2009; Troy, Kipgen, & Pal, 2015; Zipper, Qiu, & Kucharik, 2016) and are expected to continue increasing in frequency and severity in the future (Mann et al., 2018; Prein et al., 2017; Wuebbles et al., 2017).

Drought and excessive rainfall ranked, among extreme events, as the first and second largest cause of maize production loss in the United States, totaling damage of 18 and 10 billion US dollars, respectively, from 1989 to 2016 (crop insurance data from Risk Management Agency [RMA], Figure S1). The impacts of drought and heat on crops have been extensively studied using empirical‐based (Lobell et al., 2013,2014; Troy et al., 2015; Zipper et al., 2016), and model‐based approaches (Asseng et al., 2014; Deryng, Conway, Ramankutty, Price, & Warren, 2014; Glotter & Elliott, 2017; Jin, Zhuang, Tan, & Dukes, 2016). However, less attention has been paid to excessive rainfall, despite available field and experimental evidence (Hardjoamidjojo, Skaggs, & Schwab, 1982; Mukhtar, Baker, & Kanwar, 1990; Shaw & Meyer, 2015; Wenkert, Fausey, & Watters, 1981) indicating excessive water reduces crop production as often as deficient water (Hardjoamidjojo et al., 1982). The lack of quantitative studies on the excessive rainfall impact leaves a critical knowledge gap (Rötter et al., 2018), which may hinder our ability to understand and assess the climate change impacts on crops.

A similar gap exists in the development and evaluation of global process‐based crop models. Most modeling efforts, for example, the participant models in the Agricultural Model Inter‐comparison and Improvement Project (AgMIP), focus on the temperature response and CO_2_ effects on crop yield (Bassu et al., 2014; Deryng et al., 2016; Maiorano et al., 2017; Schauberger et al., 2017; Wang et al., 2017), whereas the precipitation response and the excessive rainfall impact have for some time not been in the focus (Lobell & Asseng, 2017; Rosenzweig, Tubiello, Goldberg, Mills, & Bloomfield, 2002; van der Velde, Tubiello, Vrieling, & Bouraoui, 2012). The extent that excessive rainfall adversely affects maize production in the United States is still largely unknown, especially when compared with the impact of drought (i.e., deficient rainfall), and how well current process‐based global gridded crop models (i.e., global crop models participated in the AgMIP, as opposed to point‐based models; Müller et al., 2017) simulate such impacts has not been evaluated.

In this study, we address this knowledge gap by offering new observational evidence from crop yield and insurance loss data. We first quantify the impact of excessive rainfall on US's maize production from 1981 to 2016 and compare it with the impact of extreme drought for the same period. We then investigate the regional patterns of the excessive rainfall and drought impacts and their possible explanatory factors. Finally, we compare the observed crop yield response to precipitation to that simulated by process‐based global crop models from AgMIP.

## 2 MATERIALS AND METHODS

### 2.1 Data

#### 2.1.1 Maize yield data

The county‐level maize (grain) yield and harvest area, state‐level progress report and plant population (available for limited states) data from 1981 to 2016 were obtained from the U.S. Department of Agriculture National Agricultural Statistics Service (USDA NASS, https://quickstats.nass.usda.gov/). These agricultural data by NASS are collected from multiple sources and primarily from the extensive surveys carried out during the production year, including area‐frame survey, stratified sampling farms survey, and farmer interviews (NASS, 1999). The NASS dataset is the most readily available and high‐quality crop production data in the United States and has been widely used in climate and agriculture studies (Glotter & Elliott, 2017; Schlenker & Roberts, 2009; Troy et al., 2015; Zipper et al., 2016). For the historical maize yield data, since it has a long‐term trend in yield owing to improvements in technology, seeds, and management, we estimated the linear yield trend for each county separately to calculate their detrended yield anomaly. We also tested alternative trend method (i.e., a quadratic yield trend) and the results were not affected by the methods of trend estimation.

#### 2.1.2 Crop insurance data

The county‐level crop insurance loss data from 1989 to 2016 for maize were obtained from the RMA, including “Cause of loss” (https://www.rma.usda.gov/data/cause.html) and “Summary of Business” (https://www.rma.usda.gov/data/sob.html). Cause of loss (COL) data contain indemnity amounts for different loss of causes for each year, such as “drought,” “heat,” and “excessive rainfall,” as well as the month of loss. Summary of business (SOB) data contain indemnity and premium amount, and loss ratio. The specific cause of loss (e.g., drought, heat, and excessive rainfall) is reported by farmers when they file a claim for their crop losses as required by insurance policy. The indemnity and premium amount are collected from individual cases of various insurance programs. This information is compiled by RMA to the county‐level data used in this study. Loss ratio is defined as the ratio of payments made on crop insurance policies to the total premium paid for crop insurance policies, with larger values representing greater loss. The COL and SOB data combined can give loss ratio for each loss cause (e.g., heat, drought, excessive rainfall) at specific months. The direct economic crop loss and the cause of loss information from crop insurance data provide a unique opportunity to quantitatively measure the severity of crop damage as well as attributing the damage to extreme climate. Loss ratio is used in our analysis to represent the severity of crop damage instead of the absolute indemnity amount, because loss ratio is not affected by inflation or price change over time as indemnity amount. It should be noted that crop insurance programs, their coverage, and participation rates have changed over the years during the study period (1989–2016), leading to inconsistent spatial and temporal samplings and potential biases in the analysis. However, these factors would not affect the results because our usage of the data did not rely on the temporal information and was independent of time, instead, we focused on the loss ratio of the sampled counties and their climate conditions. Therefore, the large number of county‐year samples (*N* = 46,689), though may not have complete spatial and temporal coverage, still provide enough information to derive the loss ratio across a range of climate conditions. Moreover, the loss ratio and the cause of loss data in our study only serve as independent evidence to complement the crop yield response to precipitation and the attribution to extreme climate condition.

#### 2.1.3 Climate data

Temperature and precipitation data were obtained from PRISM (Daly et al., 2008) (ftp://prism.oregonstate.edu/) at daily and 4 km spatial resolution from 1981 to 2016. The daily climate data were averaged to monthly values at each county. Maize growing season is defined from May to August. The mean growing season maximum temperature (i.e., the mean of daily maximum temperature) and the total precipitation were calculated for each county based on their monthly values.

#### 2.1.4 Tile drainage data

Tile drainage data were from the Land Use Practices data of the 2012 Census of Agriculture (https://www.nass.usda.gov/Publications/AgCensus/2012/Online_Resources/Custom_Summaries/2012_land_use_practices_by_county.xlsx). The “Land drained by tile, Acres, 2012” in the NASS 2012 census divided by the agriculture cropland area gives the percentage of cropland with tile drainage in each county.

#### 2.1.5 Soil data

The soil data were obtained from POLARIS (Chaney et al., 2016) (http://stream.princeton.edu/POLARIS/PROPERTIES/), including clay percentage and saturated hydraulic conductivity, which affect the drainage ability of soils. The soil data are available at 100 m resolution at different depths. We first extracted soil properties for maize pixels only, which were identified by the multiyear majority maize fields from the USDA Crop Data Layer data on Google Earth Engine. These county‐level soil property values were then averaged from those maize pixels from three depths up to 30 cm (0–5, 5–15, and 15–30 cm).

#### 2.1.6 Global gridded crop model simulation data

The simulation data of 12 global gridded crop models that participated in the Global Gridded Crop Model Intercomparison of the AgMIP were obtained from http://www.rdcep.org/research-projects/ggcmi (Müller et al., 2017) (hereafter the AgMIP global crop models). We used the historical simulations for maize without irrigation from 1981 to 2010, at 0.5° spatial resolution. All models were driven by the same weather dataset AgMERRA or WFDEI.GPCC with their default settings (with standard assumptions on growing seasons and fertilizer inputs). More detail on the experiment setup can be found in the Supplement of Müller et al. (2017). Following the same method of observed crop yield data, the simulated yield was first detrended and the crop yield change was calculated as the yield anomaly divided by the expected yield from their trend.

#### 2.1.7 Soil moisture data

The soil moisture dataset used is the European Space Agency Climate Change Initiative soil moisture (ESA CCI SM) v04.2 combined product (https://www.esa-soilmoisture-cci.org/). This satellite‐derived global soil moisture dataset is generated using active and passive microwave spaceborne instruments and the combined dataset of these two improves the spatio‐temporal coverage with more observation points (Dorigo et al., 2017). The dataset provides daily surface soil moisture with a spatial resolution of 0.25°, covering the period from 1978 to 2016. Due to the limitation of microwave remote sensing, the soil moisture data represent only the thin topsoil layer, that is a few centimeters in depth (<5 cm) (Liu et al., 2012). Because of the frequent gaps in the daily data, we averaged the daily data to monthly using the available data points within each month to mitigate the missing data issue (Dorigo et al., 2017). The resulting monthly data were extracted to each county to analyze the relationship between pregrowing season soil moisture and the excessive rainfall and extreme drought impacts on crop yield.

#### 2.1.8 Water storage data

The Gravity Recovery and Climate Experiment (GRACE) satellite mission, launched in 2002, collects measurements of the changes in Earth's gravity field, which can be used to derive the changes in the total land water storage (Swenson, 2012). The GRACE water storage data provide an integrated sum of changes in all vertical water components (surface water, groundwater, soil moisture, etc.) (Tapley, Bettadpur, Ries, Thompson, & Watkins, 2004). The monthly GRACE land water storage dataset used in this study was obtained from https://grace.jpl.nasa.gov/data/monthly-mass-grids/, produced by solutions of three differently institutions: GeoForschungsZentrum Potsdam (GFZ, version RL05.DSTvSCS1409), Center for Space Research (CSR, version RL05.DSTvSCS1409) at University of Texas at Austin, and Jet Propulsion Laboratory (JPL, version RL05.DSTvSCS1411). The land water storage is expressed as anomalies relative to the 2004–2009 time‐mean baseline. The data are available monthly from 2002 to 2017, at a spatial resolution of 1°. We used the average value of three solutions because the averaging can effectively reduce the noise in the data (Sakumura, Bettadpur, & Bruinsma, 2014). The water storage anomalies were extracted to each county to analyze the relationship between pregrowing season water storage and the excessive rainfall and extreme drought impacts on crop yield.

### 2.2 Define extreme climate conditions

Here, we used “standardized anomaly” (also known as standard score or z‐score) to define the degree to which climate departs from its “mean” state. Standardized anomaly can be calculated as the departure of climate of a given year ($y_{t}$) from its multiyear mean state ($\bar{y}$), normalized by the standard deviation (σ).

(1) $$y=\frac{y_{t}-\bar{y}}{\sigma }$$

We calculated standard anomaly of both growing season maximum temperature and total precipitation from 1981 to 2016 for each county, following Equation (1). Extreme drought (extreme dry) and excessive rainfall (extreme rainfall or extreme wet) conditions were defined as precipitation anomaly lower than −2σ and larger than +2.5σ, respectively (Figure S2a). The most extreme drought and rainfall conditions were defined as precipitation anomaly <−2.5σ and >+3.5σ, respectively. The uneven σ thresholds for extreme drought and extreme rainfall were chosen to ensure drought and excessive rainfall are equally represented in terms of rarity, because the precipitation distribution has a longer tail toward high precipitation. The extreme drought and extreme rainfall conditions defined this way account for roughly 1% of all county‐year samples (0.97% for extreme drought and 1.12% for extreme rainfall) during the study period (Figure S2a). Accordingly, moderate drought and moderate excessive rainfall conditions were defined as precipitation anomaly>−2σ and <2.5σ, respectively. Precipitation anomaly closer to zero is considered to be more like normal conditions (i.e., mean climate). Since this definition only considered precipitation, the identified drought and excessive rainfall were referred to as meteorological events.

Similarly, extreme heat (>+2.5σ) and extreme cold (<−2σ) conditions were defined based on standard anomaly of the growing season maximum temperature. Alternatively, extreme dry and extreme wet conditions can be defined by a given percentage (e.g., the 1% head and tail) of all county‐year samples after sorted by their precipitation standard anomaly. Through this procedure, we were able to identify the county‐specific extreme drought and extreme rainfall years based on the local climate condition of each county. This ensures that the identified extreme years represent statistically significant climate departures relative to the local climatology at each county.

### 2.3 Calculate intensity of daily precipitation

For each county, we calculated the mean and standard deviation (σ) of daily precipitation from 1981 to 2016 during the growing season period. With the mean and standard deviation calculated for each county, daily precipitation can be categorized to different intensities based on its deviation to the mean amount. For example, nine intensity categories can be defined relative to the mean daily rainfall amount following the standard anomaly approach as: <0σ, 0−0.5σ, 0.5−1σ, 1−1.5σ, 1.5−2σ, 2−2.5σ, 2.5−3σ, 3−3.5σ, and >3.5σ. The “>3.5σ” category represents the most intensive heavy rain. We calculated the contributions of each rainfall intensity to the total growing season precipitation for each county and each year (Figure S3).

### 2.4 Quantify extreme climate impacts on crop yield

By identifying the county‐specific extreme years, the impact of extreme climate year on crop yield for a given county can be quantified by comparing the crop yield of the extreme year with that expected from the county's long‐term yield trend (i.e., linear trend). Specifically, the yield difference between the extreme year and the expected yield (Yield_i_), divided by the trend yield (Yield_trend_) gives the yield percentage change Equation (2). This yield percentage change reflects the yield departure from their long‐term trend as a result of climate variability. Therefore, it can be used as a measure to quantify the extreme climate impact, by assuming that the crop yield departure under those extreme dry and wet conditions is attributable to extreme climate.

(2) $$\text{Yield percentage change}=\left({\text{Yield}}_{i}-{\;\text{Yield}}_{\text{trend}}\right)/{\;\text{Yield}}_{\text{trend}}\times 100\%$$

**Figure 1:**
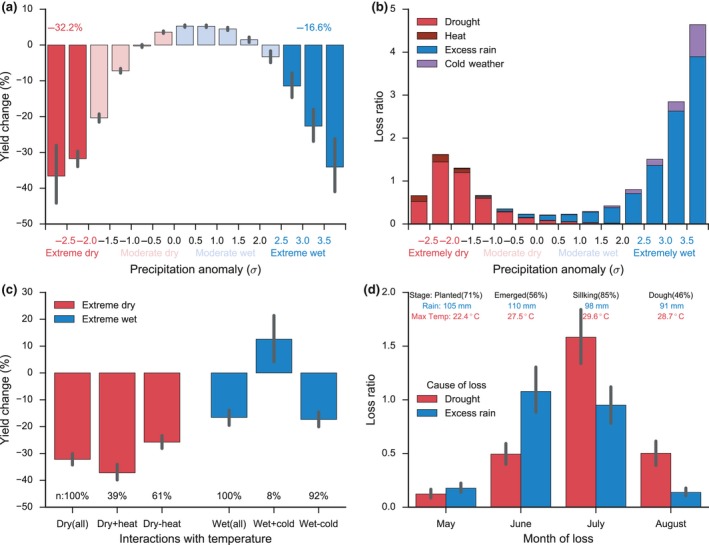
Impacts of extreme drought and excessive rainfall on maize production in the United States from crop yield (a, c) and crop insurance loss data (b, d). (a) Maize yield response to growing season precipitation anomaly from 1981 to 2016. Each bar shows the yield change weighted by harvest area from county samples in the corresponding precipitation range. The percentages shown on top are the averaged impacts of extreme drought (<−2σ, red) and extreme rainfall (>2.5σ, blue) on maize yield. (b) Crop insurance loss ratio for maize from 1989 to 2016 caused by drought, heat, excessive rainfall, and cold weather along precipitation anomaly. (c) Temperature interactions in the extreme drought and excessive rainfall impacts. Extreme drought samples (Dry [all]) are separated into drought with (Dry + heat) and without extreme heat (Dry − heat). Excessive rainfall samples (Rain [all]) are separated into extreme wet with (Rain + cold) and without extreme cold (Rain − cold). The extreme heat (>+2.5σ) and extreme cold (<−2σ) conditions are defined based on standard anomaly of the growing season maximum temperature. The percentage of the separated sample to the total sample is denoted by n. (d) Crop insurance loss ratio caused by drought and excessive rainfall in different months during extreme dry and extreme wet years. Values reported on top are the maize growth stage and its percentage from crop progress report, the monthly climatology of precipitation and max temperature from 1981 to 2016, weighted by harvest area. Error bars in panels a, c, and d denote the 95% confidence interval estimated from 1,000 times of bootstrap

The crop yield percentage change can be calculated for each county from 1981 to 2016. After obtaining the yield percentage changes as well as their corresponding precipitation anomalies for each county, the average yield impacts of extreme climate (i.e., at the national level) can be obtained by aggregating all county‐year samples that experienced extreme conditions (with precipitation anomaly of <−2σ and >+2.5σ for extreme drought and extreme rainfall years, respectively) to a weighted average yield percentage change (by county's harvest area). Also, the average crop yield response to precipitation spanning from extreme drought to extreme rainfall conditions can be obtained by aggregating county‐year samples that fall into the corresponding precipitation anomaly bins at an interval of 0.5σ (Figure 1a). The yield percentage change for each bin is the weighted average value (by county's harvest area) of county‐year samples within that bin. Similarly, the average yield response to temperature (Figure S4), and the average loss ratio across precipitation gradient (Figure 1b) can be obtained accordingly. The resulting crop yield response to maximum temperature shown in Figure S4 closely resembles the nonlinear temperature effects found in (Schlenker & Roberts, 2009), suggesting the robustness of our method. For individual county, the average extreme climate impact can be calculated as the average yield percentage change over the identified extreme years, weighted by their harvest area.

### 2.5 Compare observed yield response with simulated response from crop models

The observed crop yield percentage change (yield anomaly divided by expected yield of trend), harvest area, and bins of precipitation anomaly of each county were aggregated to 0.5° grid box to match the spatial resolution of crop model simulation. Specifically, if the center point of a county falls into a given 0.5° grid box, this county belongs to that grid box, so do their crop yield, harvest area, and precipitation anomaly bins. When one grid box contains more than one county, an area‐weighted averaged value of those counties was used for crop yield and precipitation (by harvest area of each county) at that grid box, while the sum was used for of harvest area at that grid box. The averaged responses of the observed and simulated crop yield were computed based on the 0.5 grid box, using the same area‐weighted aggregation approach as the observed county crop yield data.

## 3 RESULTS

### 3.1 The impacts of extreme drought and excessive rainfall on maize yield

Figure 1a shows the maize yield response to growing season precipitation anomaly, ranging from normal to extreme dry (<−2σ) and extreme wet (>2.5σ) conditions (see Method for definitions), over 1981 to 2006 in the United States. The maize yield percentage change is calculated as the detrended yield anomaly divided by the expected yield from the long‐term trend. The aggregated yield change from county‐year samples that experienced extreme dry and extreme wet conditions represents the impacts of extreme drought and excessive rainfall. The up to 5% positive yield anomaly observed under normal precipitation conditions was a result of favorable climate conditions for maize growth with minimal environmental stress. However, when precipitation deviated from normal toward drier and wetter conditions, the positive effect diminished and was replaced by yield losses that increased as extreme conditions intensify. The percentage of county samples exhibiting negative yield response also increased under more extreme conditions (Figure S2b). The extreme dry and extreme wet conditions, despite accounting for ~1% of county‐year samples each (Figure S2a), caused substantial damage, reducing maize yield on average by −32.2 ± 2.0% and −16.6 ± 2.7%, respectively, relative to their yield trend (Figure 1a). In particular, the most extreme rainfall (>3.5σ) caused yield reduction (−34.1 ± 7.9%) comparable to that (−36.6 ± 8.0%) of the most extreme drought (<−2.5σ). Further analysis revealed that under extreme wet conditions, the growing season precipitation was dominated by the most intensive heavy rain (>+3.5σ, see method), which contributed ~30% of total precipitation, more than any other rainfall intensities (Figure S3). These heavy rain events and associated adverse weather (e.g., hail and wind, Table S1) could cause direct physical damage to crops.

The significant yield loss under extreme drought and extreme rainfall conditions is further supported by crop insurance loss data with specified cause of loss. Here, the insurance loss ratio is defined as the ratio of payments made on crop insurance policies to the total premium paid for crop insurance policies, with larger values representing greater losses. The loss ratios for both “Drought” and “Excessive rainfall” increased significantly as precipitation departed from normal conditions (Figure 1b). Notably, under extreme conditions, the loss ratio for Excessive rainfall became even larger than Drought. In addition, the increase in loss ratios for “Heat” and “Cold weather” that were accompanied with drought and excessive rainfall revealed the close coupling of precipitation extremes and temperature.

Such interaction with temperature is particularly important for the extreme drought impact. Extreme drought coupled with extreme heat (temperature >2.5σ, 39% of extreme drought county‐year samples) resulted in yield reduction (−37.2 ± 2.9%) much greater than that without extreme heat (−25.8 ± 2.6%) (Figure 1c). This is because high temperature not only exacerbates water deficiency through increasing atmospheric water demands, that is vapor pressure deficit (Lobell et al., 2013), but also adds additional heat stress that can greatly suppress yield (Schlenker & Roberts, 2009) (Figure S4). Unlike drought, the damage of extreme rainfall seemed to primarily arise from excessive water instead of temperature interaction, since there was no clear evidence for a greater yield reduction of extreme rainfall coupled with extreme cold (temperature <−2σ, 8% of extreme rainfall county‐year samples) than that without extreme cold (Figure 1c).

Drought and excessive rainfall have varying impacts depending on the time of their occurrence relative to the crop growth stage (Daryanto, Wang, & Jacinthe, 2016; Evans & Fausey, 1999; Kanwar, Baker, & Mukhtar, 1988). Crop insurance data revealed that for extreme drought impact, July stood out as the month with the largest loss ratio (~1.5) (Figure 1d), because it coincided with a critical stage of grain filling (85% of maize in silking stage) and the hottest month of the growing season (29.6°C). These results indicate that a drought during the reproductive stage of maize, co‐occurring with heat, can cause the most damage. For the excessive rainfall impact, the largest loss ratio was found in June (1.08) and to a less extent in July (0.95) (Figure 1d). June corresponded to the early vegetative stage when most of maize was either planted (48%) or emerged (50%), which was also the wettest month of the growing season (110 mm), whereas July corresponded to the reproductive stage (silking, 85%). Field evidence suggests that both stages are susceptible to excessive moisture, especially the former period (Carter, Halverson, Rogers, & Musgrave, 1990; Kanwar, 1988).

### 3.2 The regional patterns of extreme drought and excessive rainfall impacts

**Figure 2:**
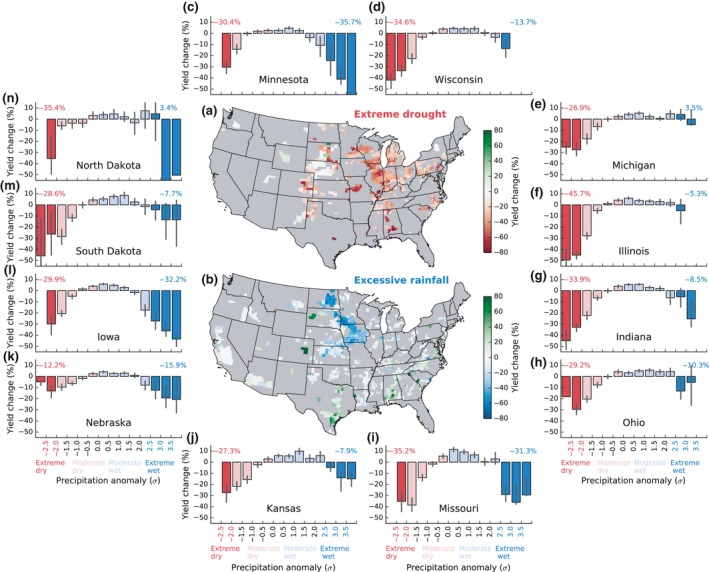
The impacts of extreme drought and excessive rainfall on maize yield from 1981 to 2016 at the county level (a, b) and in major maize production states (c–n). The extreme climate impact for any individual county on the map is the yield percentage change averaged from extreme years during the period, weighted by their harvest area (see Method for definitions). The bar chart in c–n is the same as Figure 1a but for different states

The impacts of extreme drought and excessive rainfall varied considerably across space (Figure 2a,b). Extreme drought led to consistent negative yield anomalies in most regions (in 94% of county‐year samples). The 12 major maize‐producing states in the United States (Figure 2c–n) that produced 88% of maize over the last 10 years were severely affected by extreme drought, with yield reduction ranging from −45.7 ± 3.2% (Illinois) to −12.2 ± 5.9% (Nebraska). Irrigated states (e.g., Nebraska, Kansas, Oklahoma, and Texas) generally were less affected by extreme drought than nonirrigation states (e.g., Illinois, Wisconsin, and Missouri, Figure S5).

The impact of excessive rainfall was more spatially variable and the yield response could either be negative or positive depending on the region (Figure 2b). Negative yield anomaly was observed for only 53% of county‐year samples. When including only county samples that exhibited negative yield anomaly, the averaged excessive rainfall impact would become much larger (−30.0%, Figure S6) compared to that including all counties (−16.6%, shown in Figure 1). This implies that the positive impact in some counties could cancel out the negative impact of other counties, leading to a reduced impact when averaged at regional or national scales. Spatially, the most negatively affected areas were northern states (in the Midwest), including Iowa (−32.2 ± 4.1%), Minnesota (−35.7 ± 9.7%), and Missouri (−31.3 ± 3.7%), where maize yield decrease could even exceed that caused by extreme drought. In contrast, states in the southern US still showed positive yield even under extreme rainfall conditions. The similar mixture of negative and positive yield responses was reported in China (Chen, Liang, Liang, Liu, & Xie, 2019; Chen, Liang, Liu, Jiang, & Xie, 2018), suggesting the complexity of excessive rainfall whose impact on crop yield varies in sign.

### 3.3 Comparing extreme climate impacts between observations and simulations

**Figure 3:**
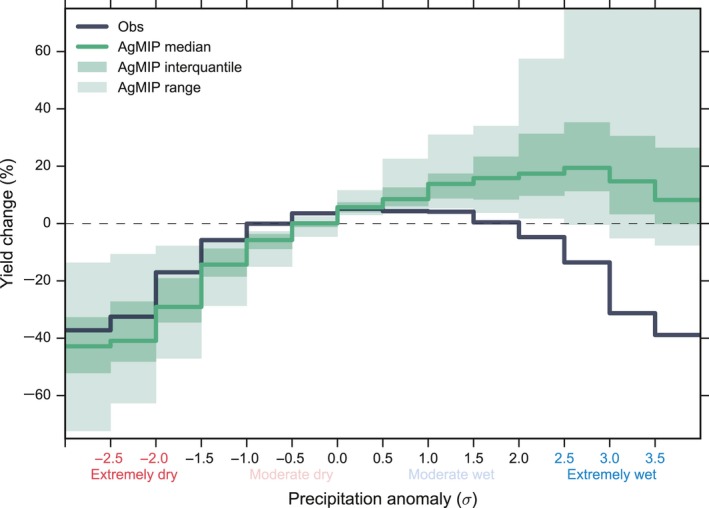
Maize yield response to precipitation anomaly from simulations of 12 global crop models participated in the AgMIP (green solid line and shaded area) compared with observed response (black solid line). Green solid line shows the multimodel median response and the shaded area shows the models interquartile range. The AgMIP global crop model data are from the historical simulations of 1981 to 2010 without irrigation, driven by AgMERRA with default setting. Same figure for individual model response is shown in Figure S7. Same figure for simulations driven by WFDEI.GPCC is shown in Figure S8

The negative yield response to drought was well reproduced by the ensemble median of 12 AgMIP global crop models (Figure 3). This well‐reproduced drought impact was partially contributed by models’ good performance in simulating the maize yield response to temperature (Figure S9) because of the high occurrence of drought and heat, as well as the strong direct relationship between drought stress and biomass (van der Velde et al., 2012). However, most crop models cannot capture the observed nonlinear yield response to precipitation and the yield reduction under excessive rainfall; instead, they showed either a reduced yield increase or increasingly higher yield with precipitation even under extreme wet conditions (Figure S7), and underestimated the percentage of negative yield response than observations (Figure S10). These results were robust to the forcing data used to drive the model simulations (alternative forcing WFDEI.GPCC results are shown in Figure S8). Only a few models (CLM‐Crop, pDSSAT, PEGASUS) showed yield decrease under the most extreme rainfall but with a much smaller magnitude. The inability of current models to simulate maize yield damage by excessive rainfall may lead to yield overestimation under wet conditions and thus bias models’ future predictions.

## 4 DISCUSSION

### 4.1 The large‐scale explanatory factors for the regionally varying impacts

**Figure 4:**
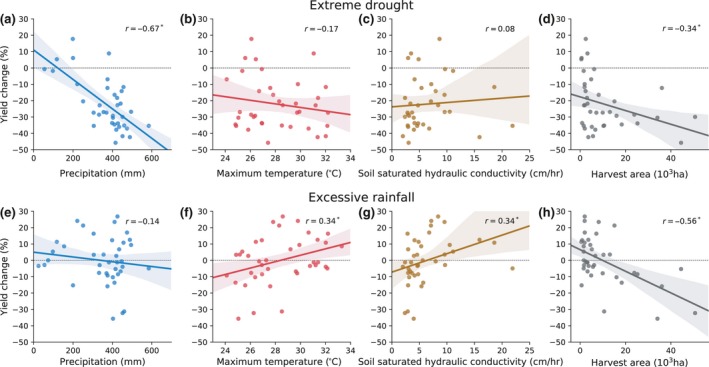
The relationship of the large‐scale climatic, edaphic, and agricultural factors with the (a–d) extreme drought and (e–h) excessive rainfall impacts on maize yield across states. Each dot represents the averaged impact of extreme drought or excessive rainfall in one state. Precipitation and temperature are the growing season mean climate from 1981 to 2016 of each state, weighted by harvest area. Soil saturated hydraulic conductivity is the weighted value up to depth of 30 cm. Harvest area of each state is the averaged county harvest area from 1981 to 2016. The solid line is the best‐fit line and shaded area is the 95% bootstrap confidence interval (*n* = 1,000). *R* is the correlation coefficient, with an asterisk denoting significance at 95%. Same figure but at the county level is provided in Figure S11 which showed qualitative similar results

The regionally varying responses of the extreme drought and excessive rainfall impacts could be explained by large‐scale factors related to mean climate conditions (growing season mean precipitation and temperature), soil (drainage characteristics) (Trnka et al., 2014), and agricultural practices (maize harvest area) (Figure 4). The spatial variation of the extreme drought impact across states was primarily regulated by precipitation (*r* = −0.67, *p* < 0.05, Figure 4a) and to a less extent by temperature (*r* = −0.17, *p* > 0.05, Figure 4b). Drought produced a much larger yield loss in wet areas than in dry areas, while there was only a weak tendency toward greater yield loss in hotter regions. The lack of impact in very dry areas is because maize is irrigated in those regions and thus less sensitive to rainfall deficiency. As expected, the drought impact decreased with a higher irrigation fraction (Figure S12). Since wet areas are typically rainfed, the impact was larger there due to the susceptibility of rainfed maize to rainfall variability, and such variability was particularly large under wet climate than dry climate (Figure S13).

For excessive rainfall impact, temperature played a more relevant role (*r* = −0.34, *p* < 0.05, Figure 4f) than precipitation (*r* = −0.14, *p* > 0.05, Figure 4e). Excessive rainfall is more likely to cause damage in cold states while benefiting the crop in warm states. Since evaporation is much slower in cold states than in warm states, the excessive rain water in cold states is more likely to cause waterlogging (flooding/ponding/saturated soils) over an extended period that harms crop growth. However, high temperature in warm states could alleviate such adverse effects and make excessive rainfall beneficial to crops through fulfilling their water demand and mitigating heat stress. We only observed a weak tendency of larger yield loss in wetter regions than in drier regions (Figure 4e).

Soil was another key factor in determining the excessive rainfall impact (*r* = 0.34, *p* < 0.05, Figure 4g) but less important for the extreme drought impact (Figure 4c). Large yield loss was found in states (e.g., Iowa, Minnesota, and Missouri) with poor‐drained soil (i.e., low saturated hydraulic conductivity, *K*_sat_, and high clay fraction), while states with well‐drained soil (i.e., high *K*_sat_, and low clay fraction) were more likely to show positive yield response (Figure 4g and Figure S14). Hence, the northern states most affected by excessive rainfall are due to the combined effects of cool climate and low‐efficient drainage of soil. In contrast, southern states, even with poorly drained soil (e.g., Texas) were not affected negatively by extreme rainfall presumably because of the mitigation effect of high temperature.

In addition to the effect of soil properties on drainage, application of artificial drainage (i.e., tile drainage) is deemed to be important (Sugg, 2007) because it could mitigate the excessive rainfall induced crop yield loss. But our analysis showed that tile drainage was not a major factor determining the regional variations in excessive rainfall impact. In fact, there was a negative relationship between the percentage of tile drainage and the excessive rainfall impact (*r* = −0.48, Figure S15a), where greater yield loss occurred in states with high percentage of tile drainage (e.g., Iowa). This can be explained by the fact that states with poorly drained soil tended to build more tile drainage (*r* = −0.20, Figure S15b), otherwise, the yield loss would be even worse without additional artificial drainage. However, for states with a small percentage of tile drainage, the yield loss by excessive rainfall could either be large because of lack of artificial drainage (e.g., Missouri) or nonexistent because of well‐drained soils (e.g., Colorado). This indicates that with confounding effects from soil property and climate, the expected effect of tile drainage could not be easily revealed in the data.

Agricultural practices could also play a role. It seemed that bigger maize production states suffered more from climate extremes than smaller production states (Figure 4d,h). This might be due to bigger maize production states with a larger harvest area also had a higher seeding density especially in recent decades (Figure S16), which may lead to a greater yield sensitivity to drought (Lobell et al., 2014). Therefore, a greater yield loss is expected to occur when these regions are hit by extreme drought. However, the causes for the larger impact of excessive rainfall in bigger maize production states are not clear and require further investigation.

### 4.2 Mechanisms and modeling of excessive rainfall impact

**Figure 5:**
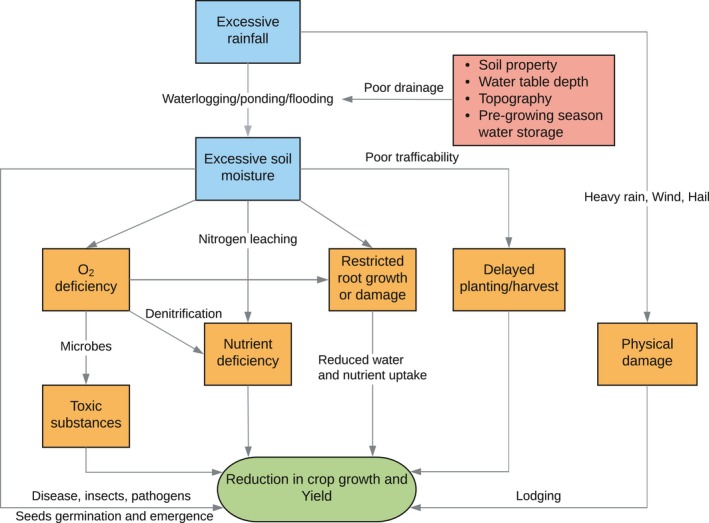
Schematic diagram of the main processes by which excessive rainfall affects crop growth and yield

In terms of mechanisms of yield loss, excessive rainfall reduces crop yield through direct physical damage and other processes associated with excessive soil water (as a result of waterlogging, ponding, flooding) that are detrimental to crops, especially under poor drainage conditions (Figure 5). The yield loss by excessive moisture can develop from: (a) root damage or restricted root development that affects plant water and nutrient uptake (Parent, Capelli, Berger, Crèvecoeur, & Dat, 2008; Wenkert et al., 1981); (b) nitrogen deficiency due to leaching or denitrification (Jabloun, Schelde, Tao, & Olesen, 2015) and the development of toxic substances, both caused by lack of oxygen in the soil (Evans & Fausey, 1999; Kanwar et al., 1988); (c) delayed planting/harvest due to poor trafficability and damage to young plants (Urban, Roberts, Schlenker, & Lobell, 2015) (e.g., seed germination and emergence). Despite these direct effects of excess water, crop can also be harmed by the (d) adverse weather events accompanied with excessive rainfall such as heavy rain (Table S1), hail (Schlie, Wuebbles, Stevens, Trapp, & Jewett, 2019), and wind (Botzen, Bouwer, & Bergh, 2010) that result in lodging; and (e) increased susceptibility to disease, insects, and pathogens (Hardjoamidjojo et al., 1982; van der Velde et al., 2012). How well crop models simulate the crop yield impact of excessive rainfall depends on the accuracy of representation of these processes within the models and the general capability in simulating soil hydrology (e.g., soil water dynamics and water table depth) and biogeochemistry (e.g., soil nitrogen dynamics). The underestimated excessive rainfall loss by crop models in Figure 3 probably reflects the lack of representation of relevant processes in the model to account for stresses related to excessive moisture (Rosenzweig et al., 2002; van der Velde et al., 2012).

While currently very few models include complete representation of these excessive soil water processes (Shaw, Meyer, McNeill, & Tyerman, 2013), there have been studies demonstrating that the crop yield response to excess water can be effectively improved by implementing empirical relationship of varying complexities, such as the stress‐day approach (Kanwar, 1988), damage function of root growth (Rosenzweig et al., 2002), or the three‐stage empirical representation of waterlogging (Shaw & Meyer, 2015). The continuous development of field‐level agronomic crop models against experimental data also paves the way for modeling these new processes, and the recent efforts to combine the strengths of agronomic crop models and global‐scale ecosystem/land surface models under a unified model framework could potentially benefit simulation of excessive rainfall impact through improved soil hydrology and photosynthesis processes (Peng et al., 2018). In addition, statistical crop models, as an independent tool to process‐based crop models, are also useful to assess the excessive rainfall impact (Lobell & Asseng, 2017; Urban et al., 2015), although their ability depends on the specific model configuration and training data (Li et al., 2019).

### 4.3 Challenges in detection and attribution of excessive rainfall impact

This study provides, to our knowledge, the first assessment of the relative impacts of excessive rainfall and extreme drought on maize yield in the United States. It is still challenging to quantify the impact of excessive rainfall on crop yield, especially under nonextreme conditions, because crop yield is an outcome of various biotic and abiotic processes integrated during the growing season. Attribution of excessive rainfall impact on crop yield can be complicated by other confounding factors. Focusing on extreme conditions (e.g., extreme wet or dry years) when crop yield significantly departs from its trend can improve detection and attribution of extreme climate impacts. However, doing so also makes the quantification inherently dependent on the definitions and occurrence of such events. A more “strict” definition of extreme event gave a larger impact on maize yield (Figure S6), because the impact is strongly dependent on the severity of extreme events. The anomalous dry years such as 1988 and 2012, and wet year 1993 which caused exceptionally large damage, also have strong contribution to the quantified negative impacts of extreme drought and excessive rainfall. Since the occurrence of such events was rare in nature and highly variable across space (Figure S17) and time (Figure S18), their impacts could be influenced by the spatial and temporal domain in which they were quantified (Figure 2), especially for excessive rainfall as it could have both positive and negative impacts on crops. Despite such dependency, our findings regarding the relationships between the excessive rainfall impacts that varied regionally and their climatic, edaphic, and agricultural factors in Figure 4 are robust to certain extreme years such as 1993 (Figure S19).

Although the impact of excessive rainfall is most prominent under extreme wet conditions, it does not mean its impact is limited to those extreme conditions. Excessive rainfall can have a wide range of influence on crop yield even under nonextreme conditions. This was evidenced by the reduced maize yield under moderate wet conditions relative to normal conditions (Figure 1a) and the presence of excessive rainfall‐induced crop loss even under normal conditions from the insurance data (Figure 1b). In contrast to the strong and long‐lasting adverse impact of drought that usually leads to a significant county‐level yield drop, the adverse impact of excessive rainfall under nonextreme cases is often realized as lower‐than‐expected yield instead of a large yield departure. This weaker yield signal is partially because the occurrence of excessive rainfall and its impact are more localized than drought, involving local factors such as soil properties (Lobell & Asseng, 2017), topography, and water table depth. Excessive rainfall loss affected more counties than drought according to insurance data, but for each case, the affected area and the damage caused, on average, were smaller than that of drought (Figure S1). Such localized features make the excessive rainfall loss recorded in the crop insurance data (which is aggregated from individual loss claim from farmers), not necessarily reflected in the county‐level crop yield data because the signal, if not large enough, gets diluted in the average yield of the whole county. Similarly, those short‐duration excessive rainfall events that are not large enough to create a strong signal in growing season, could be underrepresented in our framework, and their impacts on crop yield, therefore, can be potentially underestimated. Nevertheless, crop insurance loss data that include all conditions of crop yield loss (i.e., from both nonextreme and extreme climate conditions) can provide a more complete picture of the extent and degree of excessive rainfall impact, to complement the crop yield‐based analysis. However, uncertainties exist in the crop insurance loss data, especially for the cause of loss.

### 4.4 Pregrowing season effects of soil water storage

Our analysis is primarily based on precipitation during growing season, it should be emphasized that water storage of soil preceding the growing season may also play a role in the impacts of excessive rainfall and extreme drought on crops. By analyzing satellite‐derived ESA soil moisture and GRACE water storage data (Methods), we found that higher pregrowing season soil moisture and water storage exacerbated the yield loss by excessive rainfall while it alleviated the loss by extreme drought in the subsequent growing season (Figures S20 and S21). The relationship was strong for the impact of excessive rainfall and appeared in more pregrowing season months (from January to April) than for the impact of extreme drought (only in April). These linkages indicate that the impacts of excessive rainfall and extreme drought reflect the dynamic interactions among precipitation, soil water dynamics, and crop response.

Our results reveal that excessive rainfall, which has been largely under‐studied previously, can adversely affect maize yield as much as extreme drought, especially at regional scale. It is not only the major cause of crop damage currently in the United States for maize, but also has broad impacts for other staple crops (soybean and wheat, Figure S1), and will play a more important role in the future given the projected significant increase of extreme rainfall (Prein et al., 2017). All these call for our improved understanding and modeling of excessive rainfall impact, which is pivotal not only to provide accurate predictions of climate change impacts on agriculture but also to develop effective management and adaptation measures (e.g., drainage or levee system, cultivars (Mäkinen et al., 2018)) to mitigate the crop yield loss.

## AUTHOR CONTRIBUTION

Y.L. and K.G. conceived and designed the study; G.D.S. provided the idea and guidance for the crop insurance data analysis. Y.L. performed the data analysis; Y.L., K.G., and B.P. analyzed the results; Y.L. and K.G. wrote the manuscript with contributions from G.D.S, E.D., and B.P.

## DATA AVAILABILITY

All data used in this study are publicly available. The processed data needed to reproduce this study are available at Figshare (https://doi.org/10.6084/m9.figshare.7581473).
