# Supplementary material for: Excessive rainfall leads to maize yield loss of a comparable magnitude to extreme drought in the United States
Source: Glob Chang Biol. 2019 Apr 29;25(7):2325–37. doi: 10.1111/gcb.14628 (PMC6850578; doi:10.1111/gcb.14628)
Supplement: Supplementary file 1 [file GCB-25-2325-s001.pdf]

Table S1. The number of county-year samples of insurance loss caused by excessive rainfall and other co-occurred causes from Wind, Hail, and Flood from 1989 to 2016 for maize. Of the 26,563 county-year samples for excessive rainfall loss, 23.6%, 28.5% and 27.4% are associated with loss caused by Excess wind, Hail, and Flood, respectively. Note that the county-year samples for causes of loss by Wind, Hail, and Flood have overlaps due to the co-occurrence.

Excess Moisture/Precip/Rain (n=26,563)	Wind/Excess Wind	23.6% (n=6,262)
	Hail	28.5% (n=7,559)
	Flood	27.4% (n=7281)

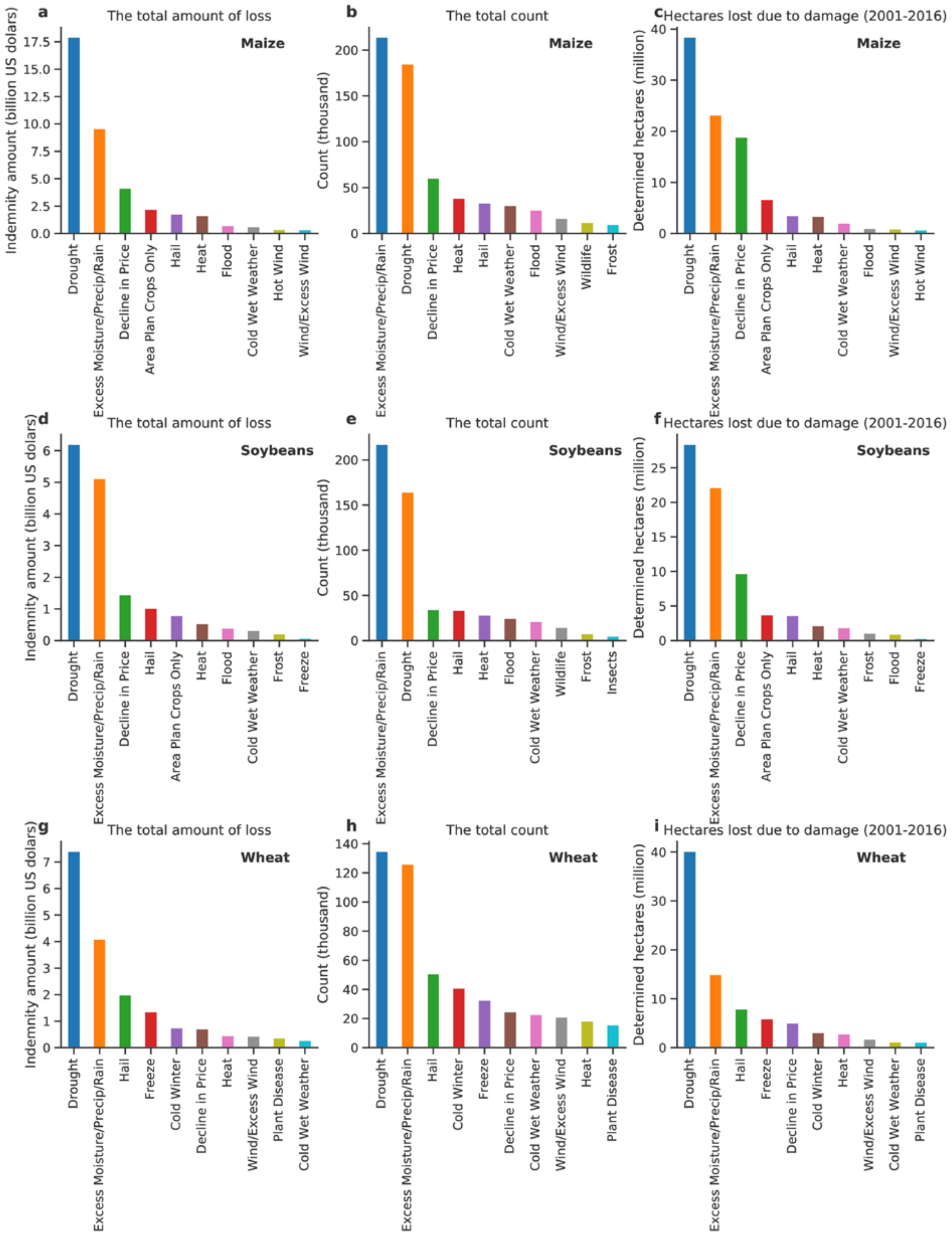

Fig. S1. The top ten causes of crop loss for maize, soybeans, and wheat in the US from the RMA insurance data. (a,d,g) The total amount of loss (sum of indemnity amount) from 1989 to 2016. (b,e,h) The total count of loss causes from 1989 to 2016. (c,f,i) The total hectares (sum) lost due to damage from 2001 to 2016.

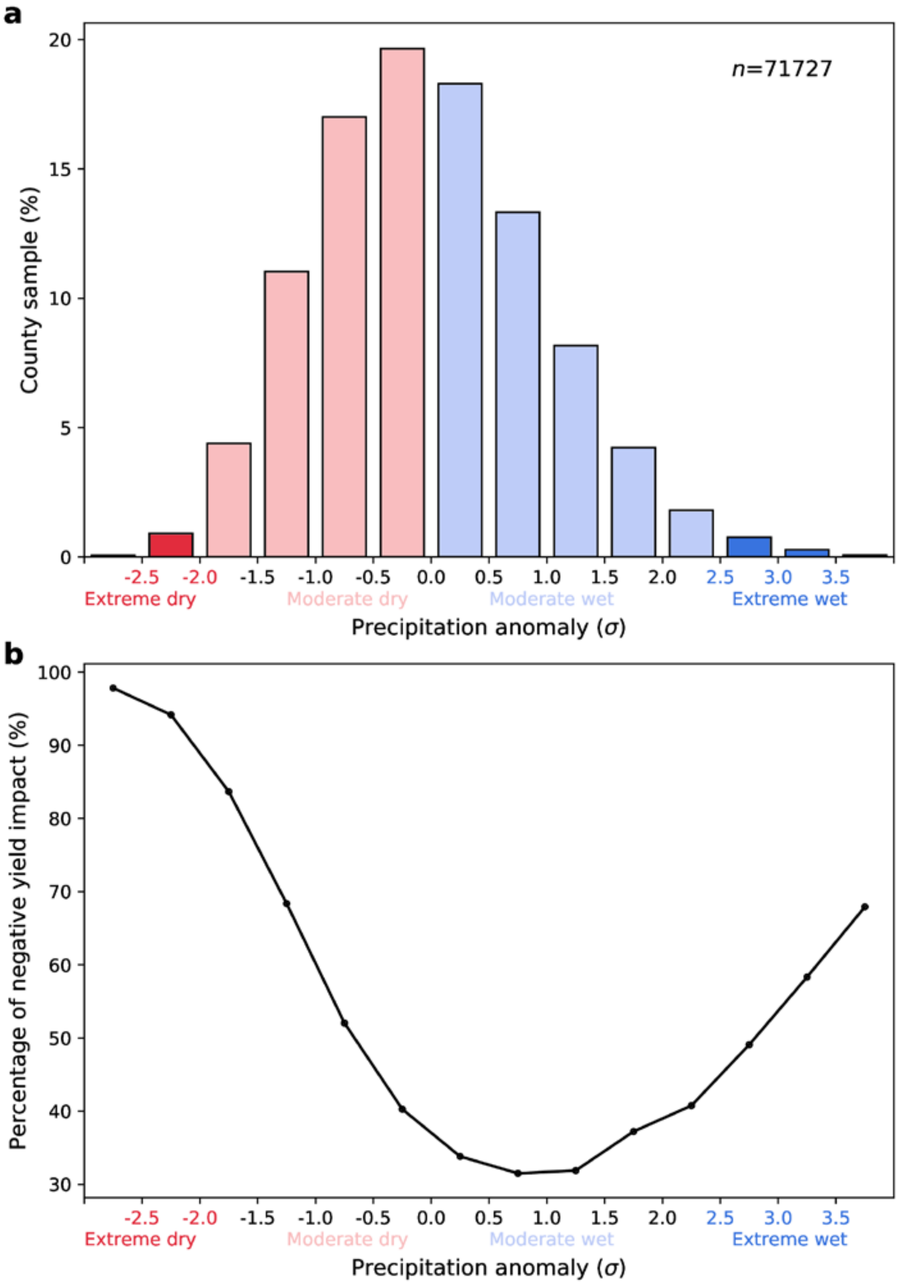

Fig. S2. (a) The number of county-year samples contained in each precipitation anomaly bin (%). The total number of county sample is 71,727. (b) The percentage of county samples exhibiting negative yield anomaly in each precipitation anomaly bin.

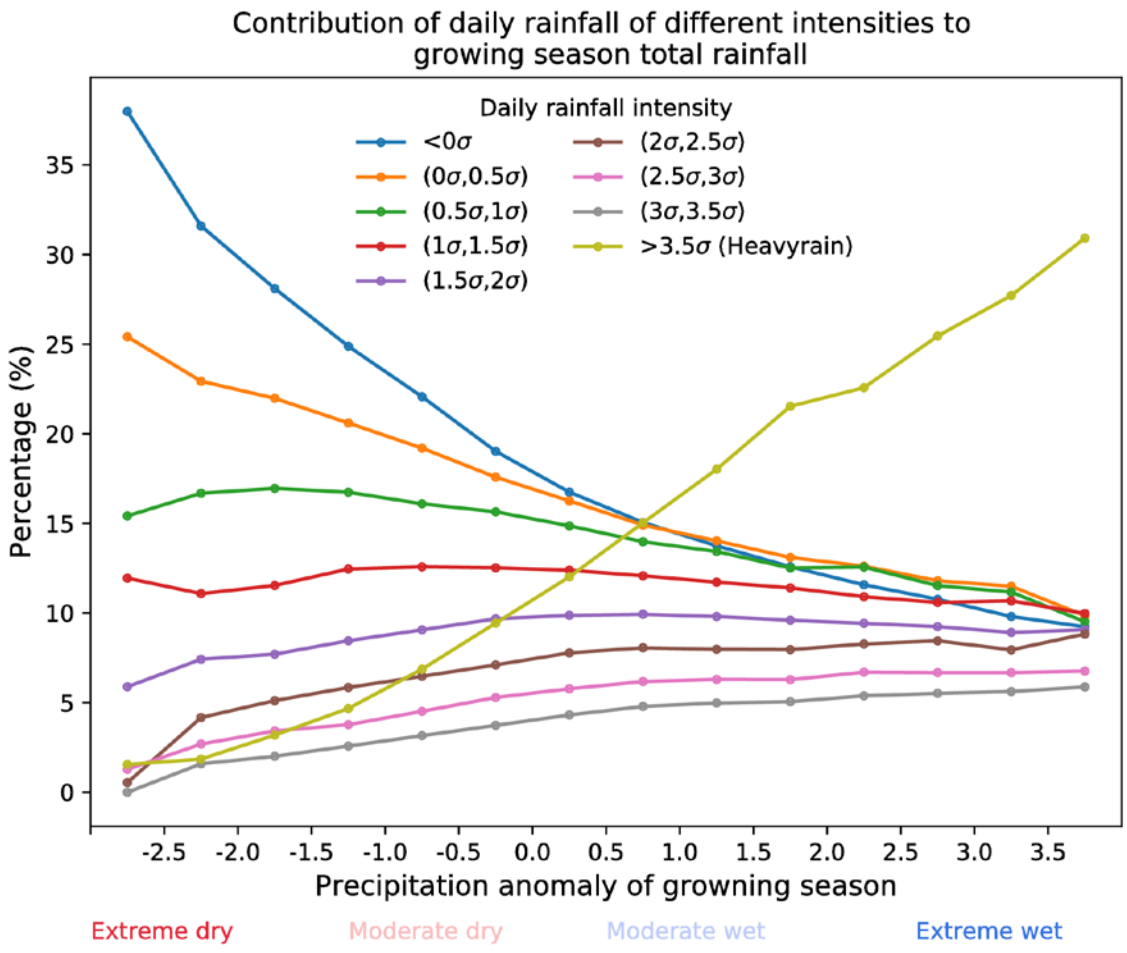

Fig S3. The contributions of different rainfall intensities to the growing season total precipitation from extreme dry to extreme wet conditions, averaged from 1981 to 2016. The rainfall intensity is defined based on the standard anomaly of daily rainfall (i.e., <0σ, 0–0.5σ, 0.5–1σ, 1–1.5σ, 1.5–2σ, 2–2.5σ, 2.5–3σ, 3–3.5σ, and >3.5σ, see method) and the “>3.5σ” category represents the most intensive heavy rain.

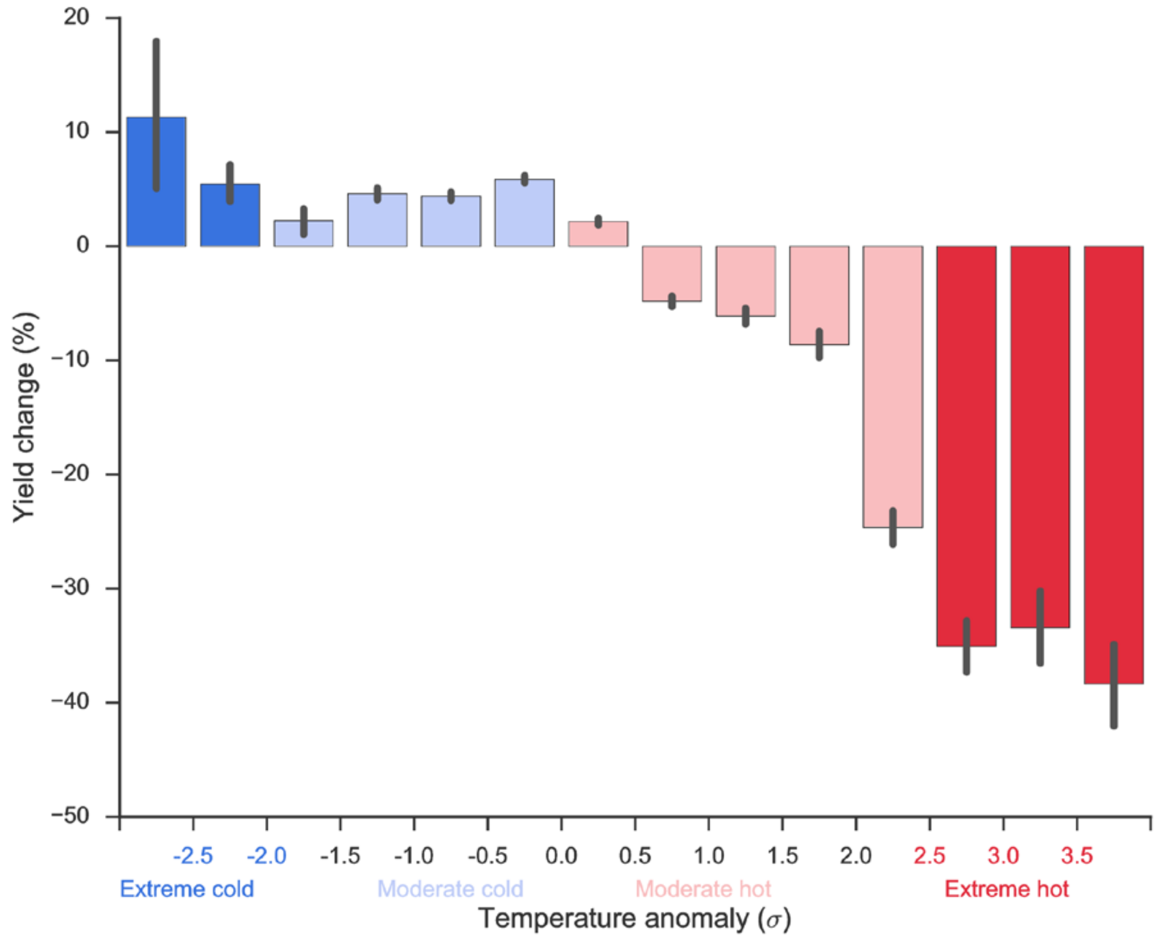

Fig. S4. The observed maize yield response to maximum temperature anomaly from 1981 to 2016. Error bars denote the 95% confidence interval estimated from 1,000 times of bootstrap.

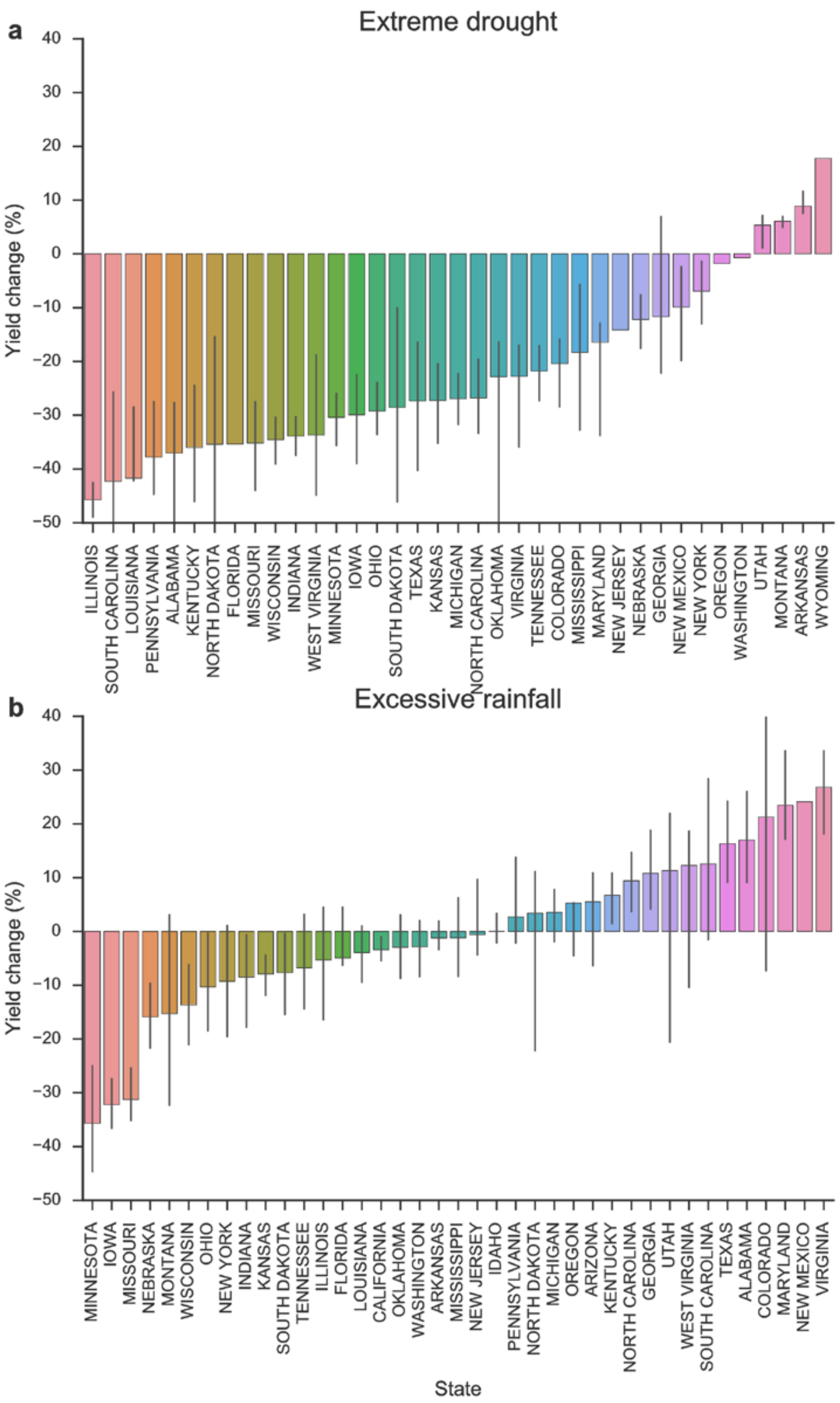

Fig. S5. The impacts of extreme drought (a) and excessive rainfall (b) on maize yield from 1981 to 2016 for individual states. Error bars are estimated from 1,000 boot strapping at 95% significance level.

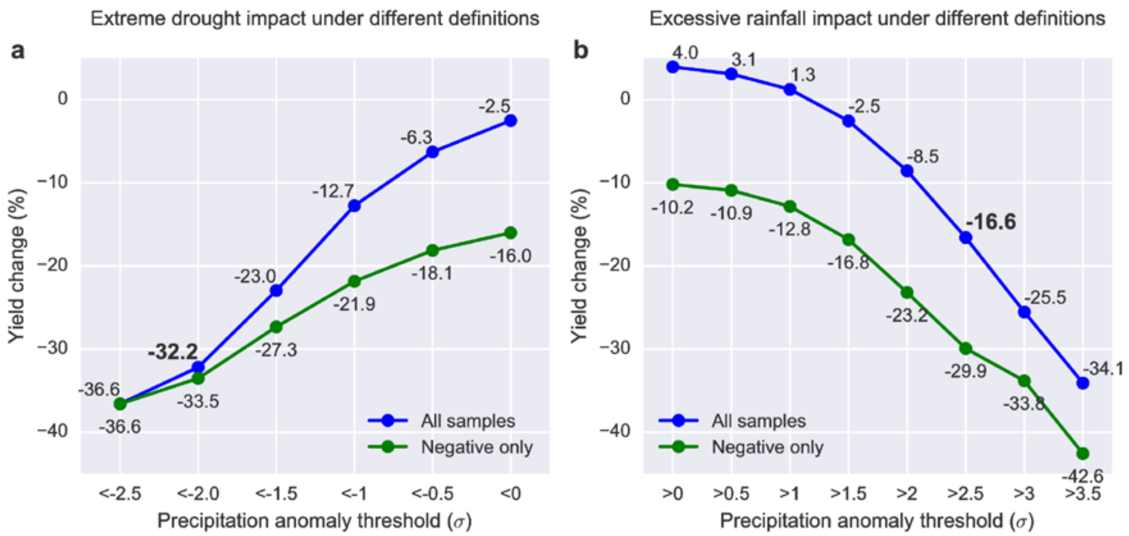

Fig. S6. The extreme drought (a) and excessive rainfall (b) impacts on maize yield vary with their definitions. Extreme drought and excessive rainfall are defined as standard precipitation anomaly below or above certain thresholds. Each dot shows the impacts of excessive rainfall (or extreme drought) on yield (y-axis) defined by the corresponding threshold of precipitation anomaly on the x-axis. The more "strict" definition gives larger impact. The bold font number denotes the definition we adopted to report the results in the main text. The yield impact can be calculated from including all county samples (blue line) or only including county samples that exhibit negative yield anomaly (green line). The latter shows larger impact on maize yield.

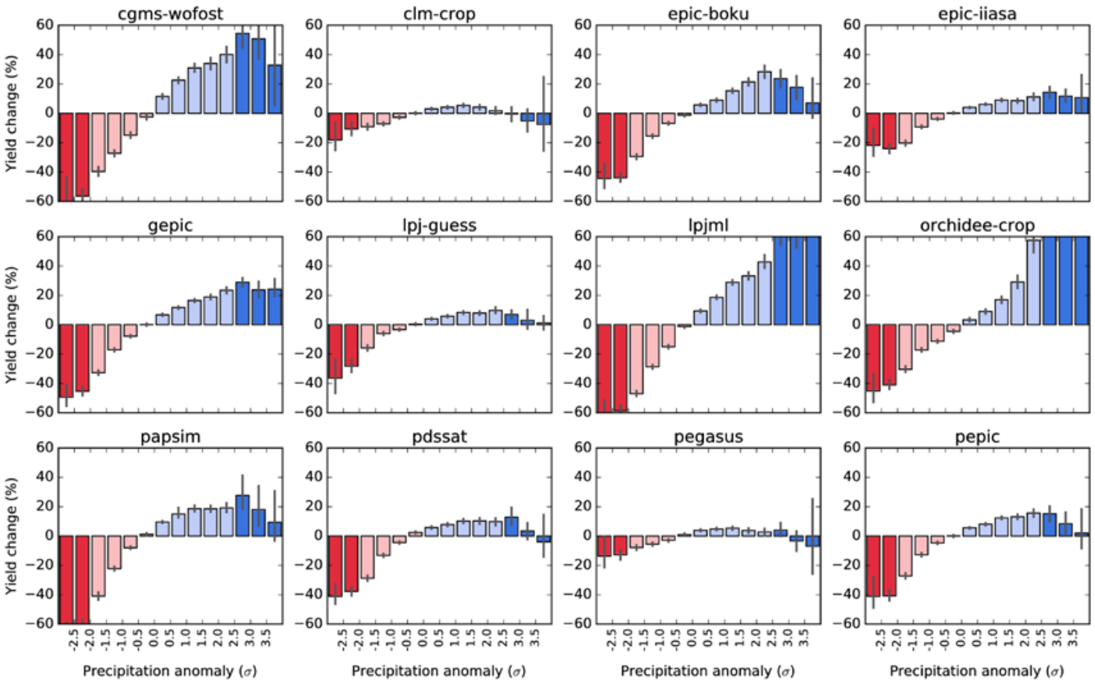

Fig. S7. The simulated maize yield response to precipitation anomaly for 12 individual AgMIP global crop models.

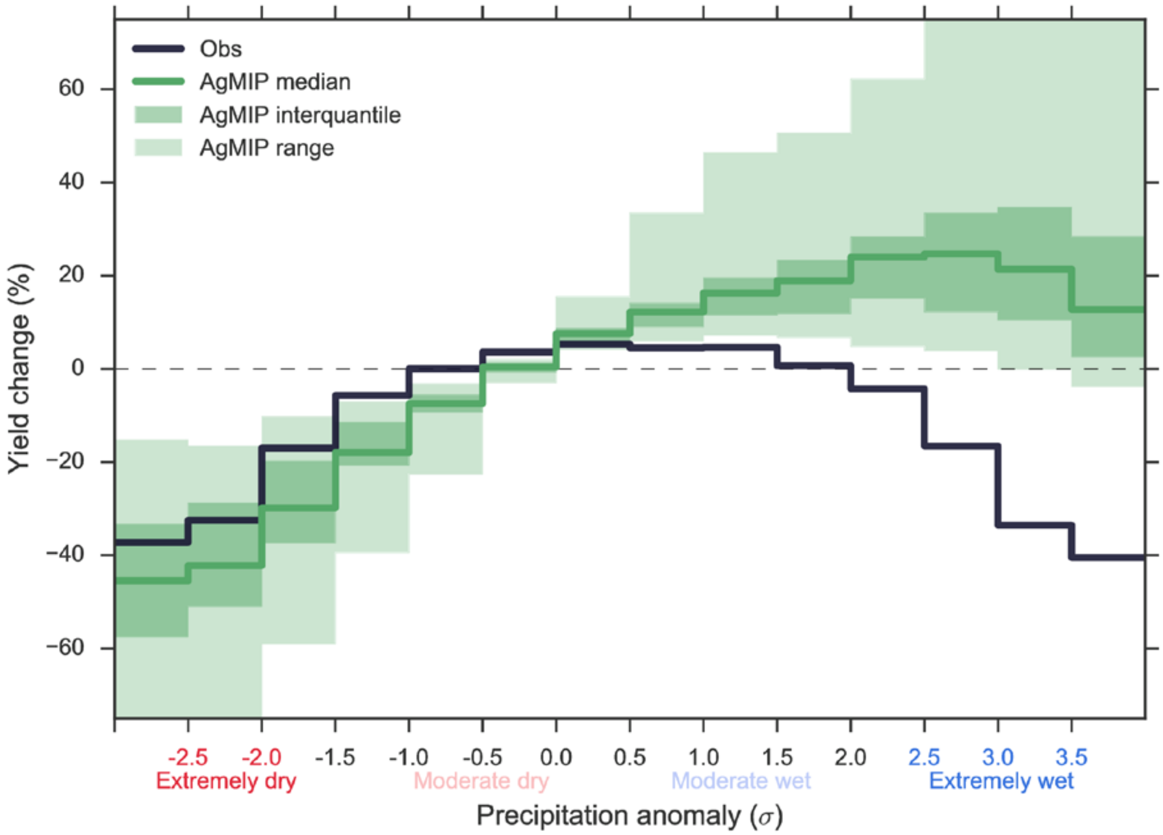

Fig S8. Maize yield response to precipitation anomaly from simulations of 12 global crop models participated in the AgMIP (green solid line and shaded area) compared with observed response (black solid line). Same as Fig 4 but the simulation is driven by WFDEI.GPCC.

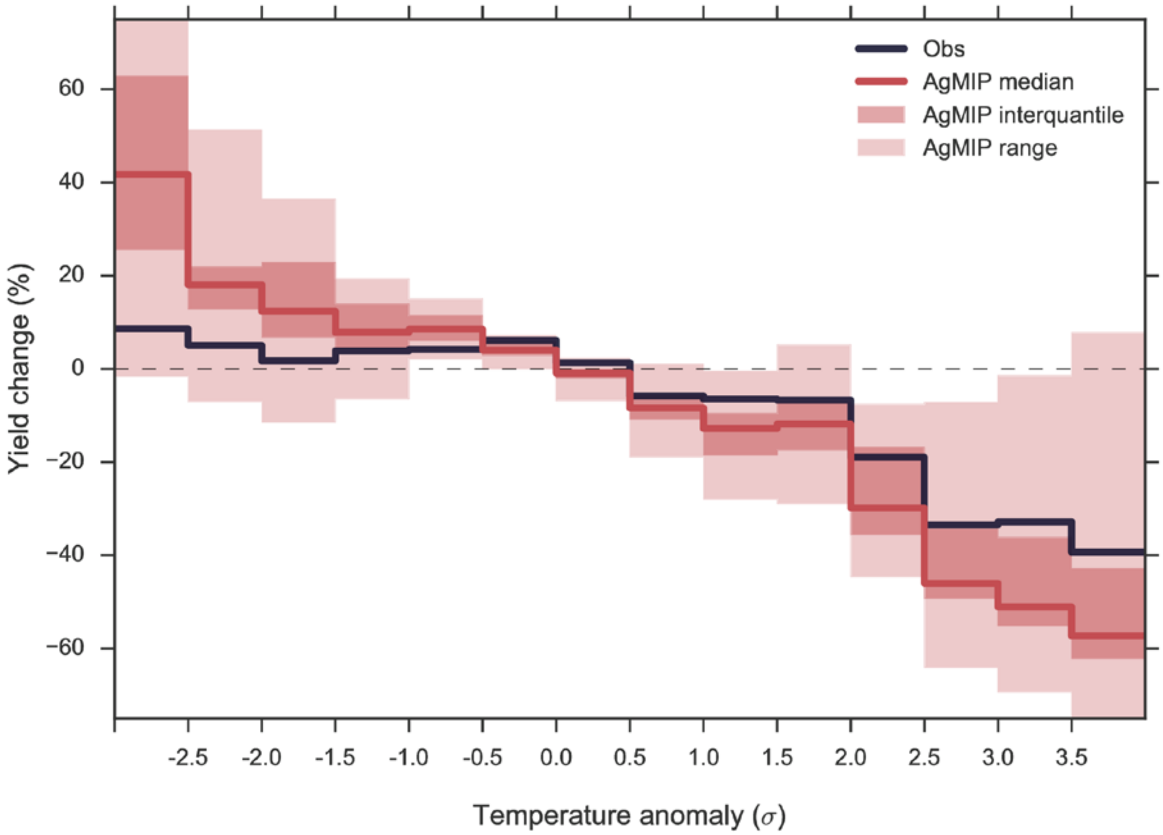

Fig. S9. Simulated maize yield response to temperature anomaly from 12 AgMIP global crop models compared with observed response. The simulation is driven by the AgMERRA forcing.

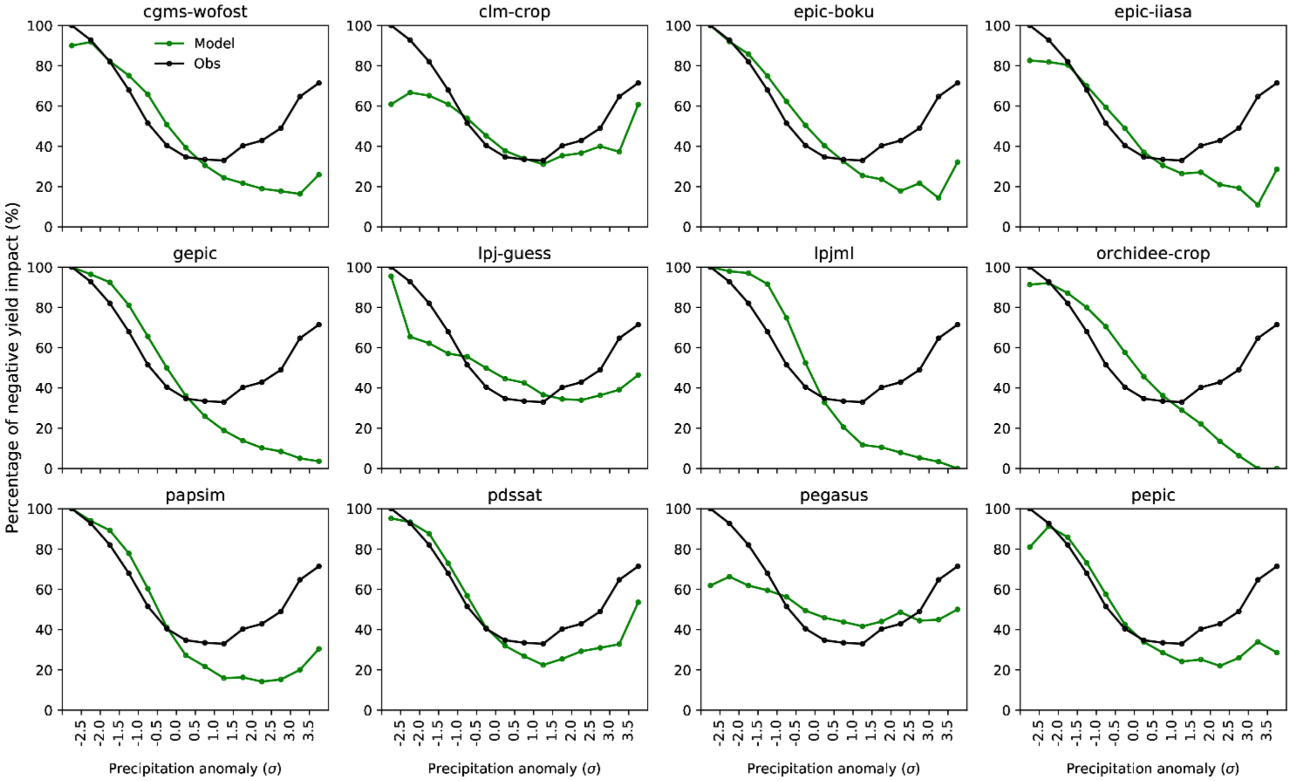

Fig. S10. The percentage of simulated yield (grid-year samples) exhibiting negative yield anomaly in each precipitation anomaly bin from 12 AgMIP global crop models driven by the AgMERRA forcing. Similar results with the alternative forcing WFDEI.GPCC are not shown.

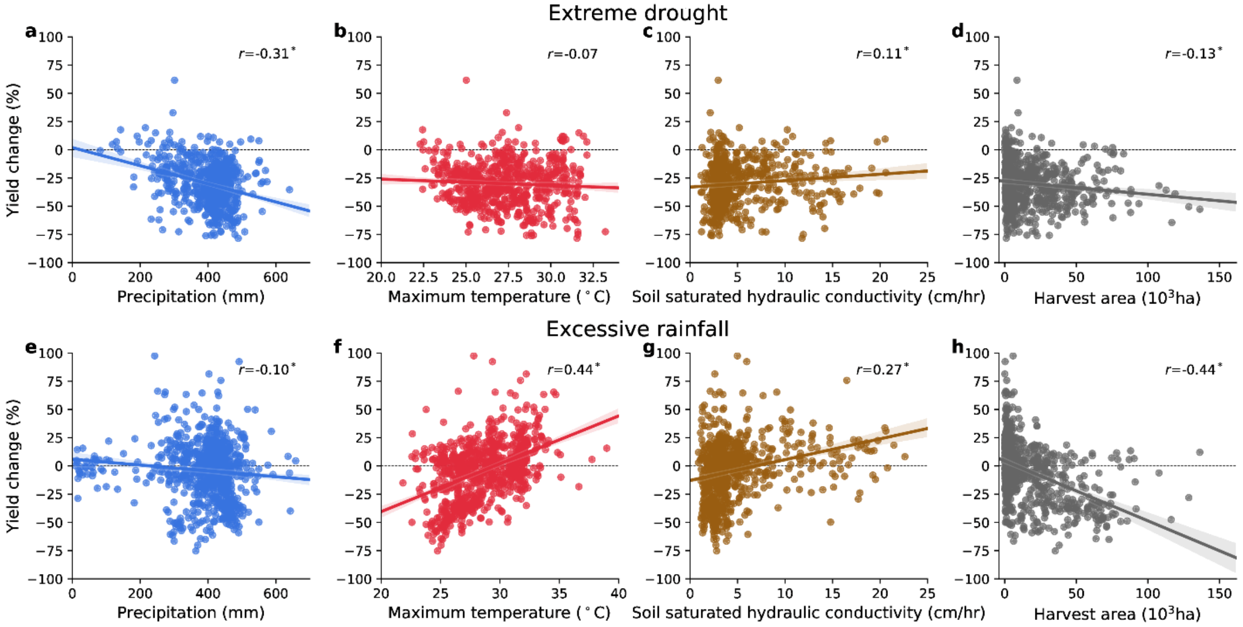

Fig. S11. Same as Fig 4 but at the county level. The relationship of the large-scale climatic, edaphic, and agricultural factors with the **(a-d)** extreme drought and **(e-h)** excessive rainfall impacts on maize yield across counties.

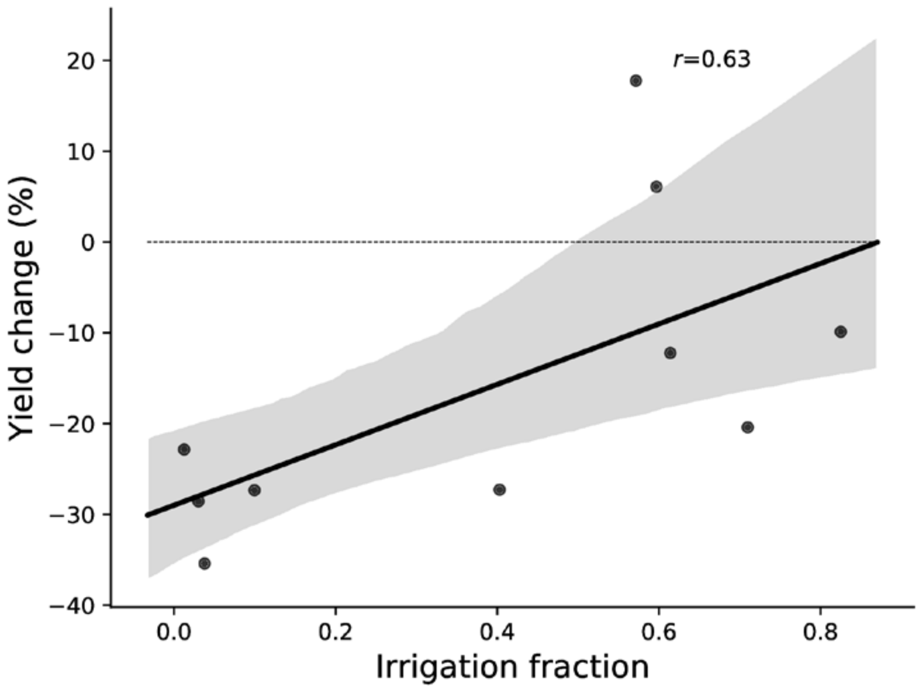

Fig. S12. The relationship between irrigation fraction and the extreme drought impact of each state. The 11 states with irrigated maize and their areal fractions are: Colorado (71%), Delaware (5%), Kansas (40%), Montana (60%), Nebraska (61%), New Mexico (83%), North Dakota (4%), Oklahoma (1%), South Dakota (3%), Texas (10%), Wyoming (57%).

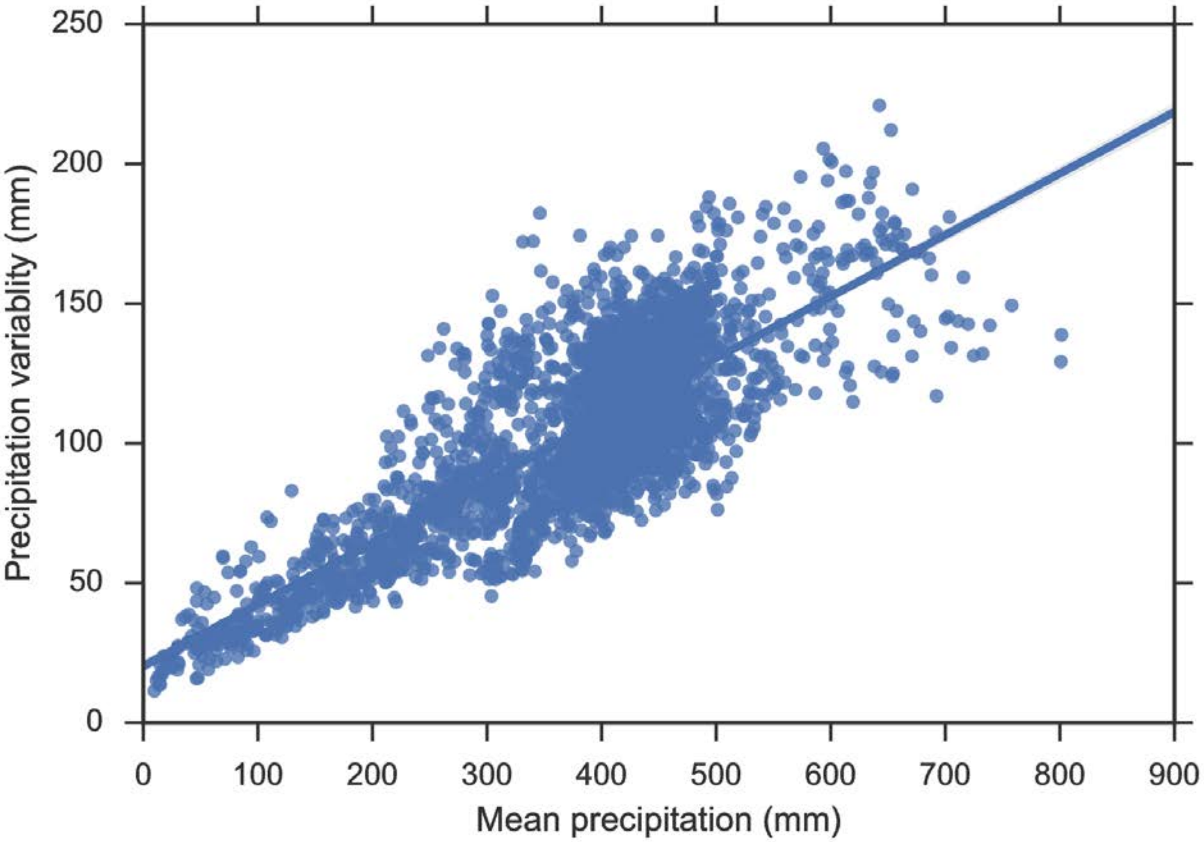

Fig. S13. Relationship between growing season mean precipitation and its interannual variability (standard deviation) using county data from 1981 to 2016. The solid line is the fitted line.

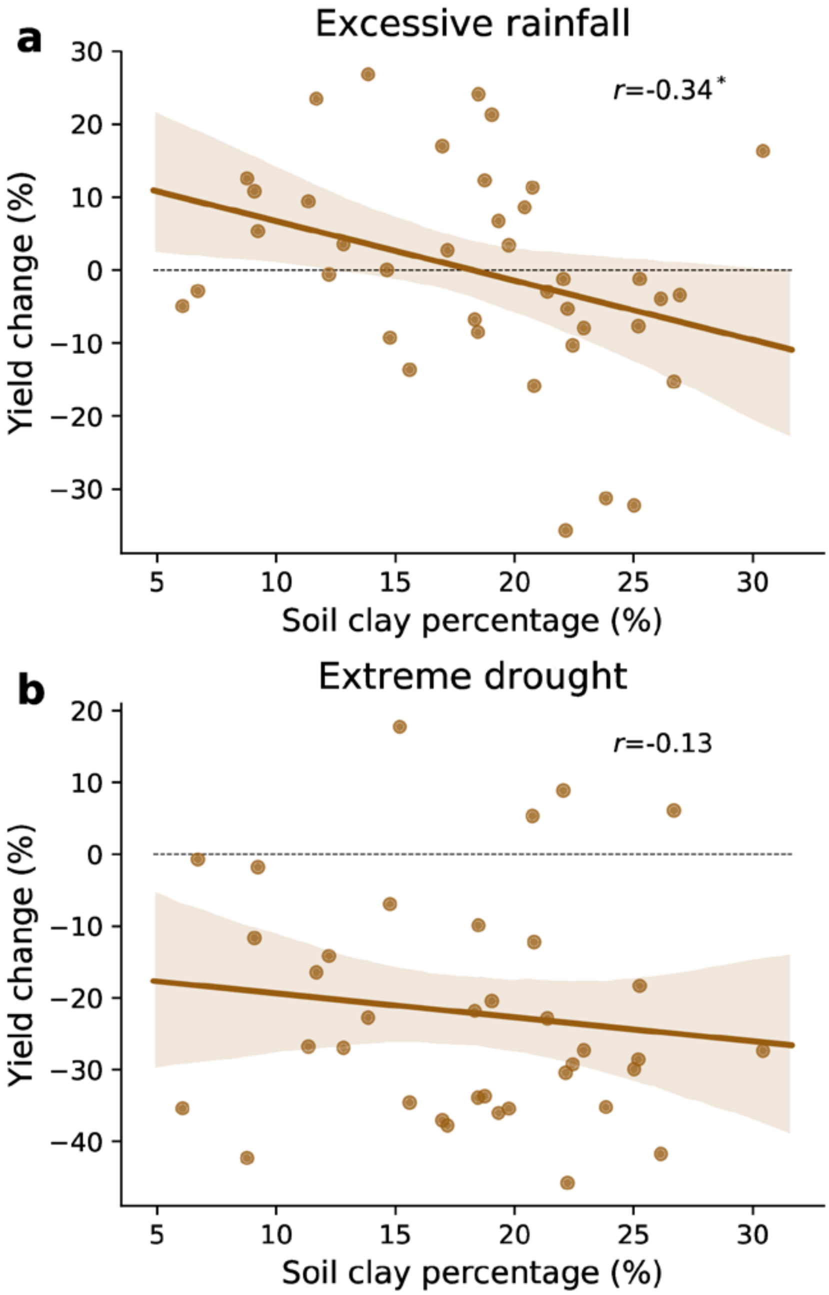

Fig. S14. The relationship between soil clay percentage and the impacts of (a) excessive rainfall and (b) extreme drought. Each dot represents the impact in one state. Soil clay percentage is the weighted value by soil depth to 30 cm. The solid line is the best-fit line and shaded area is the 95% bootstrap confidence interval (n=1,000). R is the correlation coefficient, with an asterisk denoting significance at 95%.

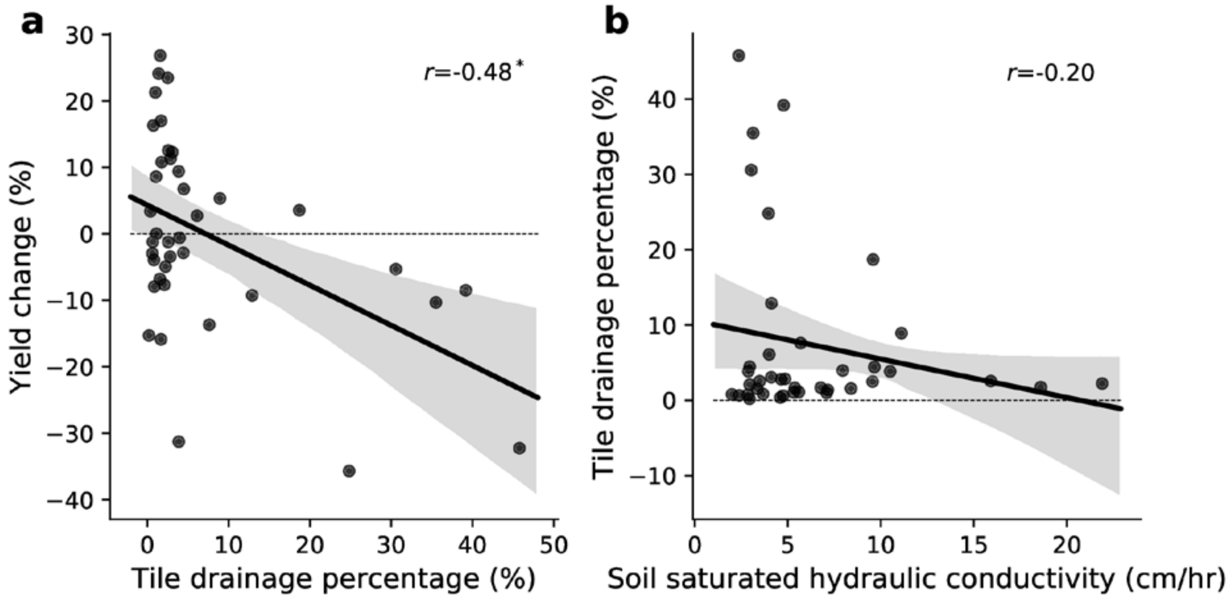

Fig. S15. (a) Relationship between tile drainage percentage and maize yield impact of excessive rainfall across states. (b) Relationship between soil saturated hydraulic conductivity and tile drainage across states. Each dot represents the impact of excessive rainfall in one state and their corresponding tile drainage percentage. The solid line is the best-fit line and shaded area is the 95% bootstrap confidence interval (n=1,000). R is the correlation coefficient, with an asterisk denoting significance at 95%.

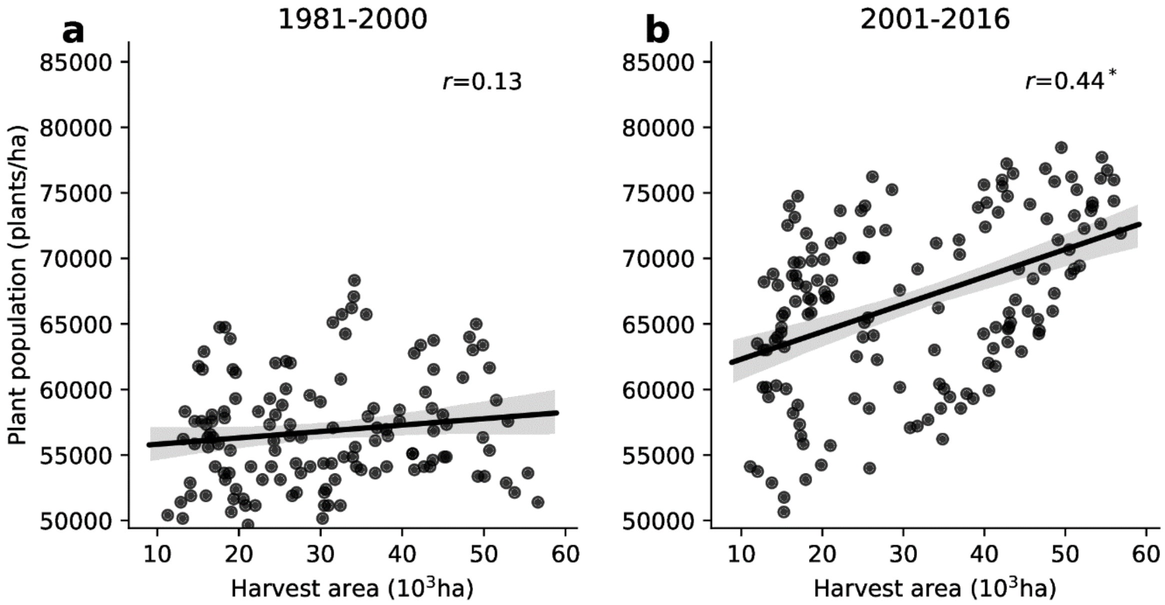

Fig. S16. Relationship between maize plant population and harvest area in selected states (a) from 1981 to 2000, and (b) from 2001 to 2016. The solid line is the best-fit line and shaded area is the 95% bootstrap confidence interval (n=1,000). R is the correlation coefficient, with an asterisk denoting significance at 95%. The state level maize population data are available for selected states including Illinois, Indiana, Kansas, Minnesota, Missouri, Nebraska, Ohio, South Dakota, and Wisconsin.

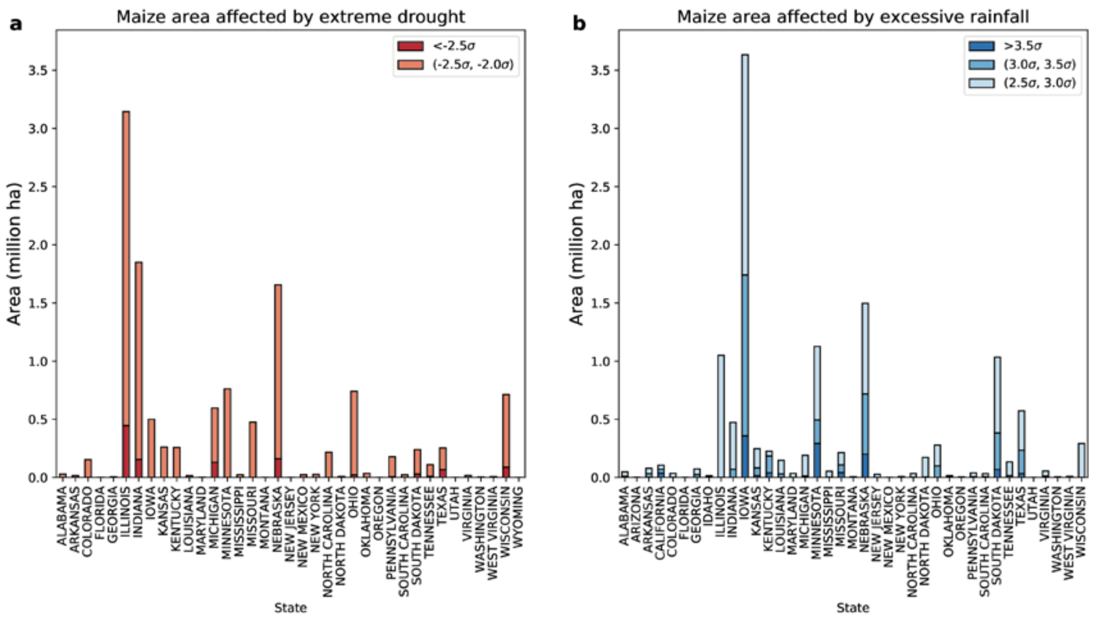

Fig. S17. Maize harvest area affected by (a) extreme drought and (b) excessive rainfall in different states from 1981 to 2016.

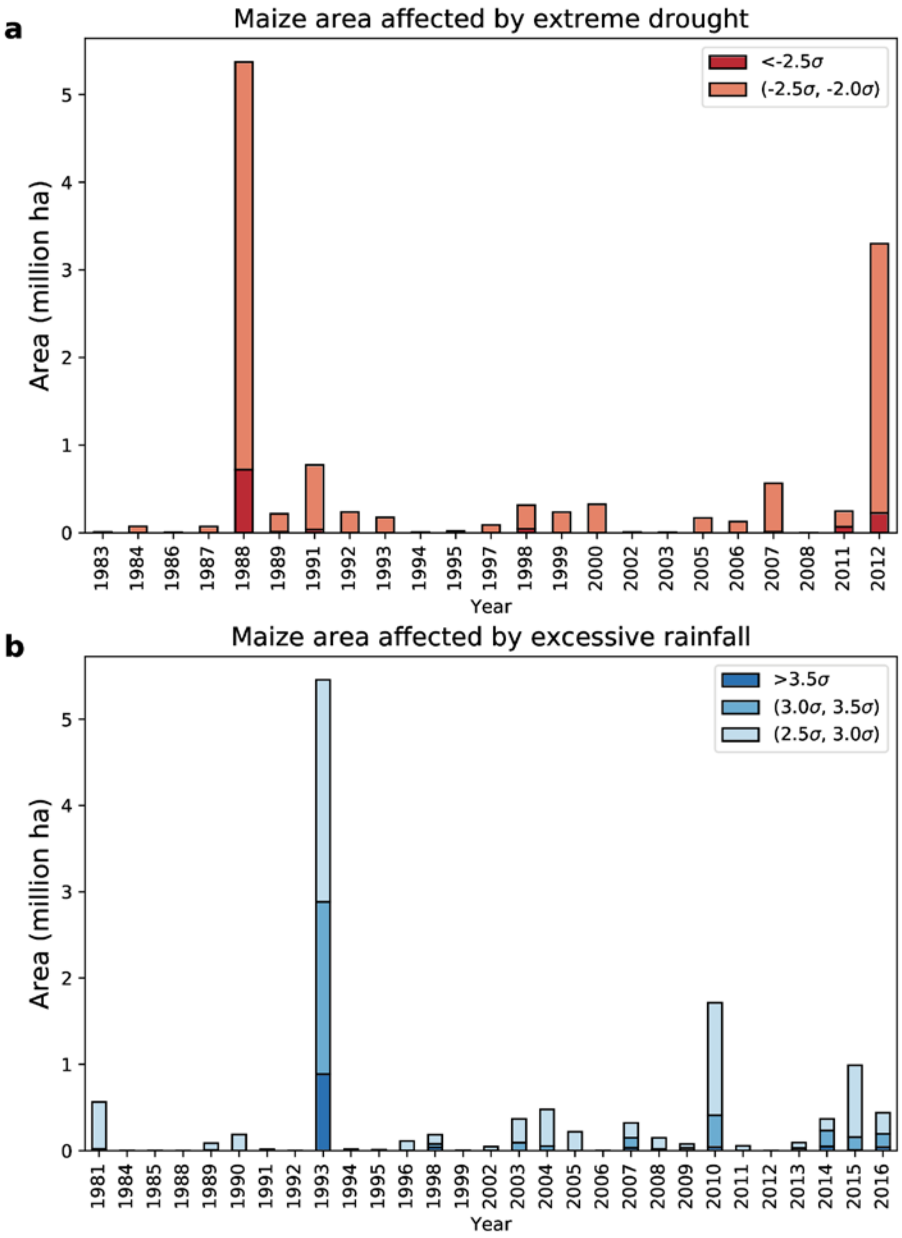

Fig. S18. Maize harvest area affected by (a) extreme drought and (b) excessive rainfall in each year during the study period.

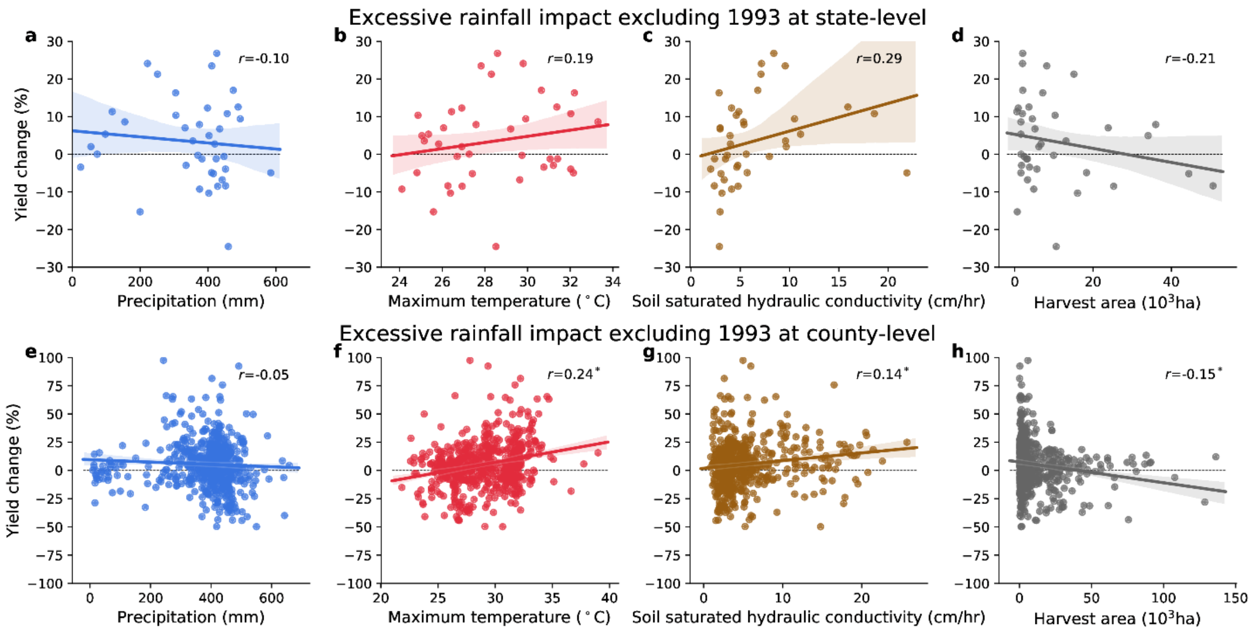

Fig. S19. Same as Figs 4 and S11 but for excessive rainfall impacts and the extreme year 1993 is excluded. The relationship of the large-scale climatic, edaphic, and agricultural factors and the excessive rainfall impacts on maize yield at **(a-d)** the state level and **(e-h)** the county level.

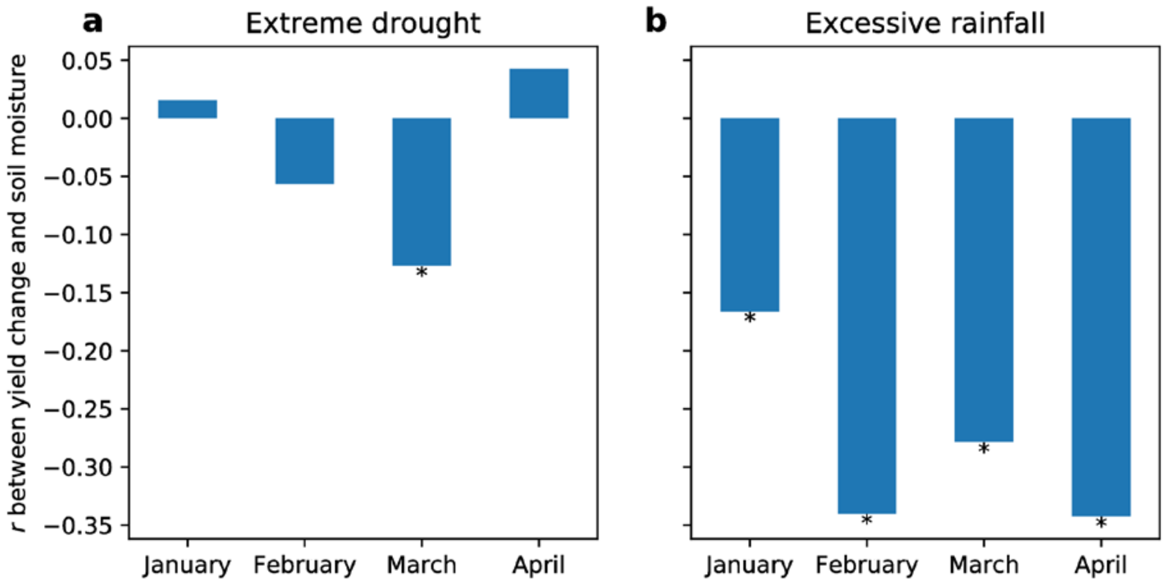

Fig. S20. Relationship between the pre-growing season soil moisture and the maize yield impacts of extreme drought (a) and excessive rainfall (b). The correlation coefficient is calculated from the yield change and their pre-growing season soil moisture from January to April, using the county-year samples classified as extreme drought/excessive rainfall from 1981 to 2016. The asterisk on each bar indicates that the correlation coefficient is statistically significant at 95%. Note that for correlation with the extreme drought impact, county samples from states with irrigation (Colorado, Delaware, Kansas, Montana, Nebraska, Texas, South Dakota, New Mexico, North Dakota, Oklahoma, Wyoming) were excluded. For correlation with excessive rainfall impact, only county samples from states that exhibited yield loss under excessive rainfall conditions (averaged from 1981 and 2016) were used.

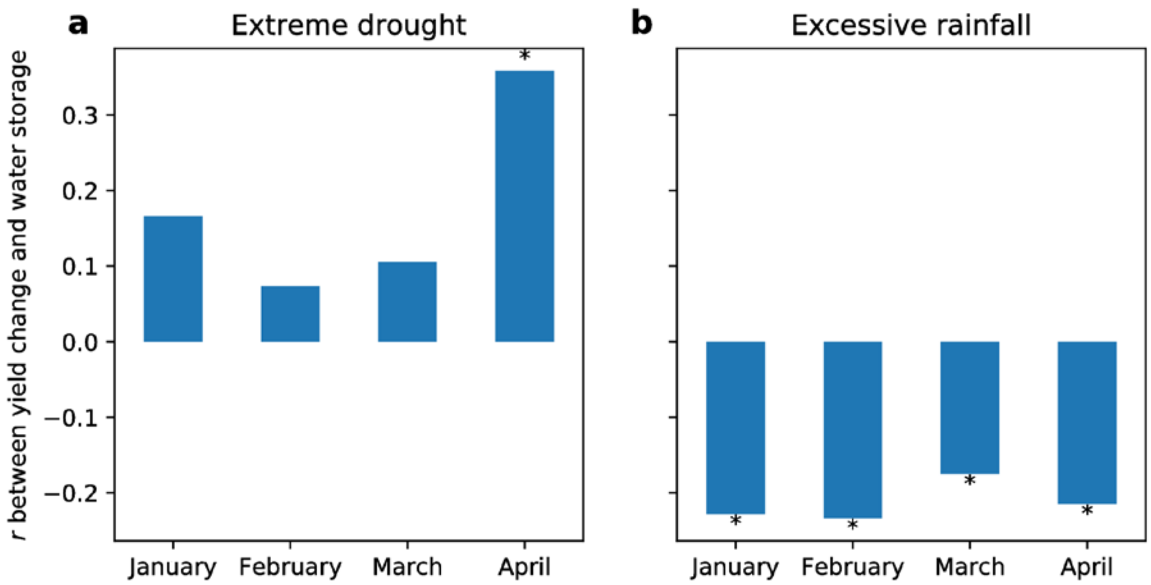

Fig. S21. Relationship between the pre-growing season water storage and the maize yield impacts of extreme drought (a) and excessive rainfall (b). The correlation coefficient is calculated from the yield change and their pre-growing season water storage anomaly fields from January to April, using the county-year samples classified as extreme drought/excessive rainfall from 2002 to 2016. The asterisk on each bar indicates that the correlation coefficient is statistically significant at 95%. Note that for correlation with the extreme drought impact, county samples from states with irrigation (Colorado, Delaware, Kansas, Montana, Nebraska, Texas, South Dakota, New Mexico, North Dakota, Oklahoma, Wyoming) were excluded. For correlation with excessive rainfall impact, only county samples from states that exhibited yield loss under excessive rainfall conditions (averaged from 2002 and 2016) were used.
